# Supplementary material for: Lying mirror using structured surfaces
Source: Nat Commun. 2026 Aug 7;17:9140. doi: 10.1038/s41467-026-76488-2 (PMC13521926; doi:10.1038/s41467-026-76488-2)
Supplement: Supplementary file 1 [file 41467_2026_76488_MOESM1_ESM.pdf]

Supplementary Information for

Lying mirror using structured surfaces

Yuhang Li[1,2,§], Shiqi Chen[1,2,§], Bijie Bai[1,2], and Aydogan Ozcan[*,1,2,3]

[1]Electrical and Computer Engineering Department, University of California, Los Angeles, CA, 90095, USA.

[2]California NanoSystems Institute (CNSI), University of California, Los Angeles, CA, USA.

[3]Bioengineering Department, University of California, Los Angeles, 90095, USA.

[§] Equal contribution

*Correspondence: Aydogan Ozcan. Email: ozcan@ucla.edu

This Supplementary Information file includes:

- **Parameters and Digital Implementation for Numerical Analyses**
- **Supplementary Figures S1-S11**

Parameters and Digital Implementation for Numerical Analyses

For the lying mirror designs operating under monochromatic coherent illumination, a numerical analysis was conducted at a wavelength of $\lambda = 0.75\ mm$. While these analyses were performed in the terahertz range, the lying mirror design is inherently scalable with respective to the illumination wavelength λ; it can be adapted to operate in different spectral ranges, including the visible part of the spectrum, by simply scaling its diffractive features according to the illumination wavelength. This scalability was demonstrated in our experimental results, which showcased the successful implementation of the lying mirror designs in the visible spectrum.

The setup included a single phase-modulation layer coupled with a standard reflective mirror. The diffractive layer consisted of 120×120 trainable diffractive features, each approximately 0.53λ in size. The axial distance between any two successive planes was set as 26.7λ. The structured mirror design shown in Fig. 7 maintained the same diffractive feature count and axial distances as the aforementioned configuration. For the Fashion-MNIST and MNIST datasets, all available training images were utilized for training of the corresponding lying mirror design. For the QuickDraw dataset, 100,000 images from the 'dog' category were selected and then divided into two sets: 80,000 for training and 20,000 for testing. The target "dummy" image was randomly chosen from the 'panda' category. During training, each input image was linearly resized to 60×60 pixels before being fed into the lying mirror design. The central 60×60 pixel region at the output FOV was cropped for the loss function calculation.

In Fig. 4, we evaluated the system's robustness by exposing the lying mirror design to various forms of random image distortions. These included random rotations within ± 90 degrees, random scaling between 50% and 200%, and random lateral shifts within ± 15 pixels (8λ) in both the x and y directions, which were achieved by built-in functions of *torchvision.transforms*. As for the image noise conditions reported in Fig. 5, random Gaussian noise (ε) was introduced to the input images. Following the addition of random noise, any negative values in the resulting images were clipped to zero.

For the experimental demonstrations shown in Fig. 8, the structured lying mirror was trained to transform input images to a target image under three distinct illumination wavelengths, i.e., $\lambda = 480, 550$, and 600 nm. We trained the structured mirror to process phase-only input images while capturing intensity at the output plane. During training, each original 28×28 grayscale MNIST image was linearly upscaled to 40×40 pixels (with a pixel size of $19\ \mu m$) using the nearest-neighbor interpolation. The structured mirror

comprised 150×150 trainable diffractive features, each with a lateral size of $10.8\ \mu m$. The axial distance between the input and the structured mirror was set at $136.8\ mm$, and the distance between the structured mirror to the CMOS camera was $145.0\ mm$. The central 40×40 pixels within the output FOV were cropped for the loss function calculation, detailed earlier. To better visualize each captured image, we selected the R, G, B channels of the captured raw images from our color camera to represent $600, 550$ and $480\ nm$ illumination results, respectively.

In the experimental demonstration shown in Fig. 9, the structured lying mirror was trained to transform its input images into a target image under broadband illumination ranging from $520\ nm$ to $570\ nm$. Following the above-outlined procedures, we trained the structured mirror to process phase-only input images while capturing intensity at the output plane. During the training, each original 28×28 grayscale MNIST image was linearly upscaled to 112×112 pixels (with a pixel size of $8\ \mu m$) using nearest-neighbor interpolation. The structured mirror consisted of 300×300 trainable diffractive features, each with a lateral size of $8\ \mu m$. The axial distance between the input and the structured mirror was set to $120.1\ mm$, while the distance from the structured mirror to the CMOS image sensor was $96.4\ mm$. The central 112×112 pixels within the output FOV were cropped for the loss function calculation.

For the simulation of a lying mirror under spatially partially coherent illumination, the wavelength was selected as $532\ nm$. During the training, each original 28×28 grayscale MNIST image was linearly upscaled to 80×80 pixels (with a pixel size of $8\ \mu m$) using nearest-neighbor interpolation. The structured mirror consisted of 200×200 trainable diffractive features, each with a lateral size of $8\ \mu m$. The axial distances were the same as those used in the broadband lying mirror designs. The central 80×80 pixels within the output FOV were cropped to calculate the time-average intensity $\hat{O}$ for the loss function calculation. All quantitative data analyses, including PCC values, PSNR values and loss function calculations, were performed on raw data without any image contrast enhancement applied.

The adversarial digital neural networks that were employed to reconstruct/transform the dummy images back into the input images, reported in Supplementary Figs. S8 and S9, were based on the U-Net architecture[1].

Multi-wavelength, broadband, and spatially partially coherent lying mirror models were trained with a batch size of 16 for 30 epochs, while all the other models were trained with a batch size of 150 for 200 epochs. The Adam optimizer was used with default parameters and a learning rate of 0.001. The layers were initialized with zero phase. The training process used PyTorch (v1.11.0, Meta Platforms Inc.) on a GeForce

RTX 3090 GPU (NVIDIA Inc.). The training process for the numerical monochromatic lying mirror models took ~2 hours, while the experimental multi-wavelength lying mirror model required ~12 hours of training.

References

1. Ronneberger, O., Fischer, P. & Brox, T. U-Net: Convolutional Networks for Biomedical Image Segmentation. in *Medical Image Computing and Computer-Assisted Intervention – MICCAI 2015* (eds Navab, N., Hornegger, J., Wells, W. M. & Frangi, A. F.) 234–241 (Springer International Publishing, Cham, 2015). doi:10.1007/978-3-319-24574-4_28.

Supplementary Figures

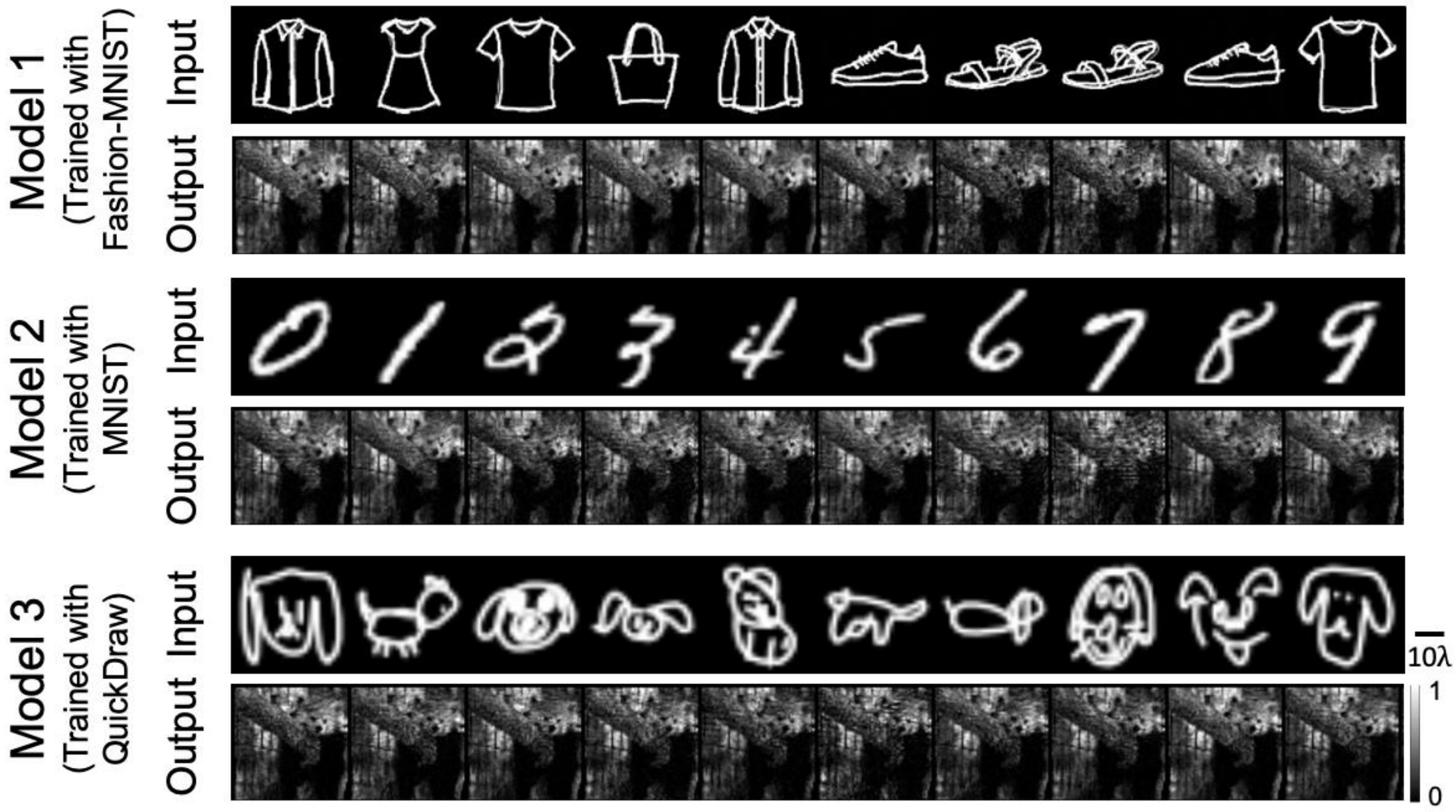

Figure S1. Blind testing results of lying mirrors. Three lying mirror models demonstrate the framework's ability to transform diverse input images from the Fashion-MNIST, MNIST, or QuickDraw datasets into an out-of-distribution output image (a panda) randomly selected from the ImageNet dataset. For publication, the original Fashion-MNIST input images are replaced with representative images depicting the same object classes to comply with copyright requirements. These input images are shown for visualization only; the original images used in our figure/analysis correspond to image indices 9900–9909 in the test split and the original dataset order was preserved without shuffling. All reported experiments, quantitative results, and the corresponding output images were generated using the original benchmark datasets described in the Methods, and are unchanged.

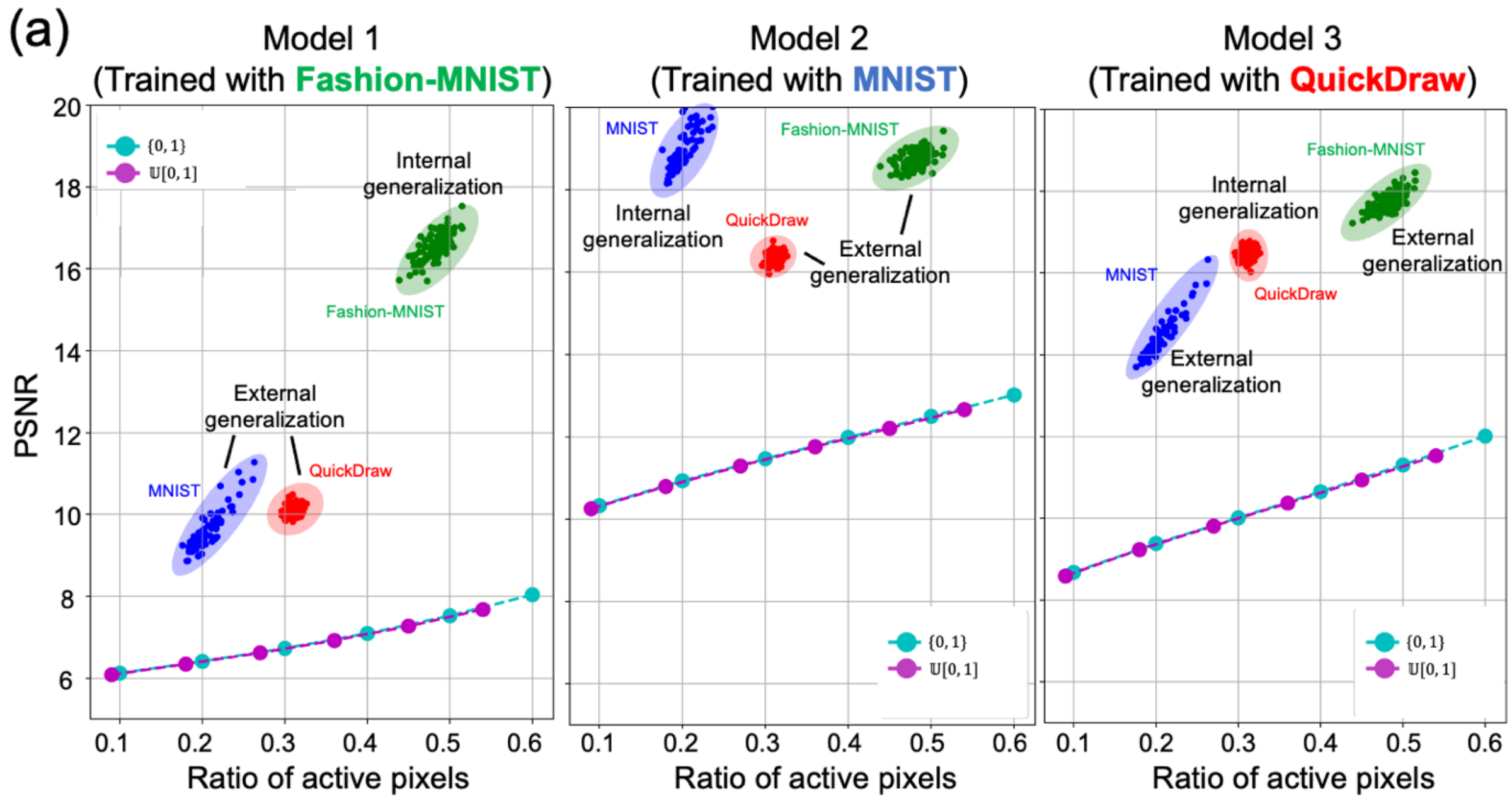

Figure S2. Quantitative analysis of the performance of lying mirror outputs for different input images. Three lying mirror models, trained with the Fashion-MNIST, MNIST, and QuickDraw datasets, were blindly evaluated using these 3 image datasets as well as binary random patterns and random patterns with uniform distribution 𝕌[0,1]. The performance of the outputs was quantitatively assessed using the PSNR metric.

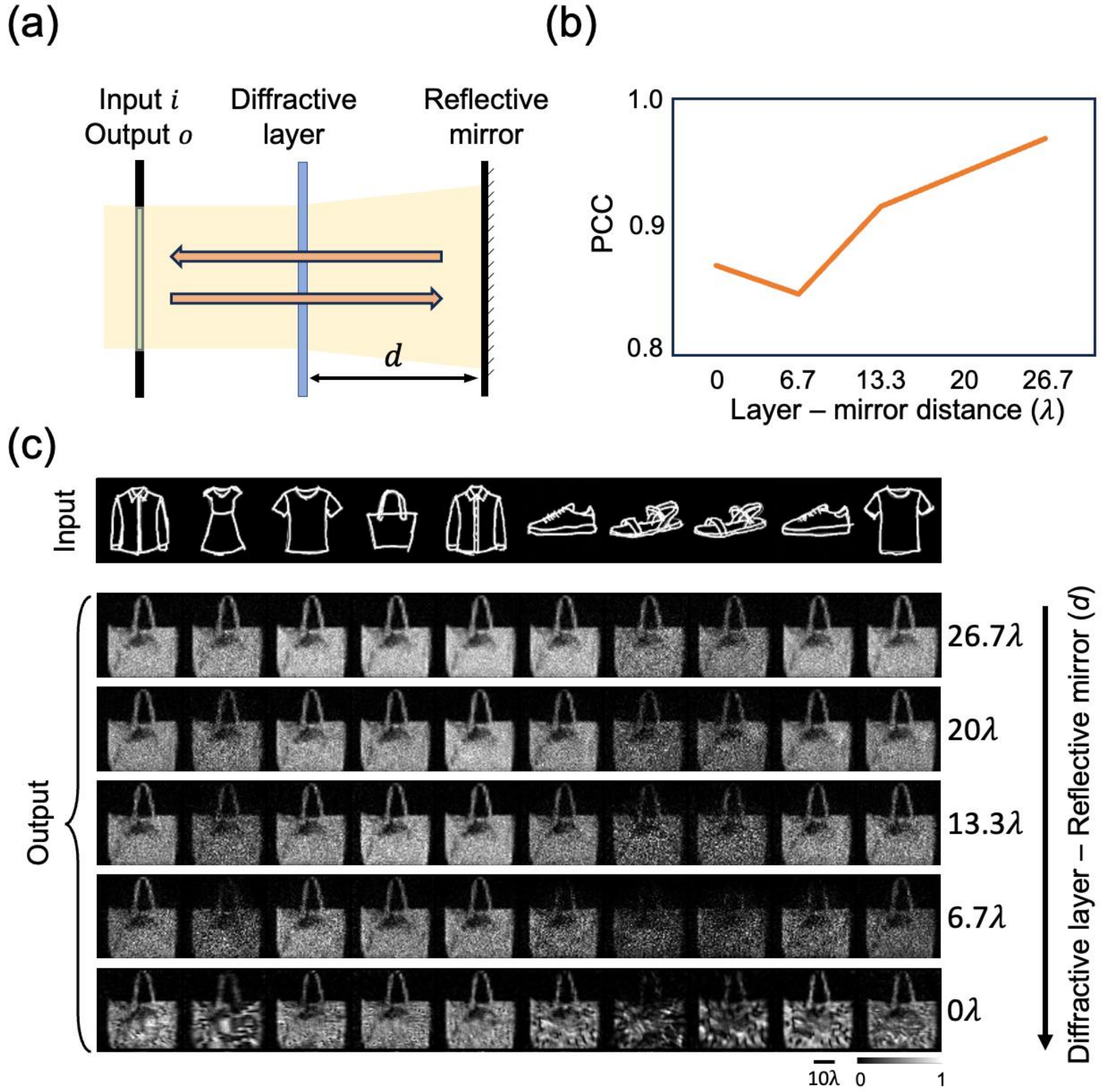

Figure S3. Evaluation of the performance of lying mirrors under various axial distances between the diffractive layer and the standard reflective mirror. (a) Schematic of a lying mirror with different diffractive layer-to-reflective mirror distances (d). (b) Quantitative performance analysis and (c) visualizations of the lying mirror outputs as a function of the axial distance between the diffractive layer and the reflective mirror. For publication, the original Fashion-MNIST input images have been replaced with representative images depicting the same object classes to comply with copyright requirements. These input images are shown for visualization only; the original images used in our figure/analysis correspond to image indices 9900–9909 in the test split and the original dataset order was preserved without shuffling. All reported experiments, quantitative results, and the corresponding output images were generated using the original benchmark datasets described in the Methods, and are unchanged.

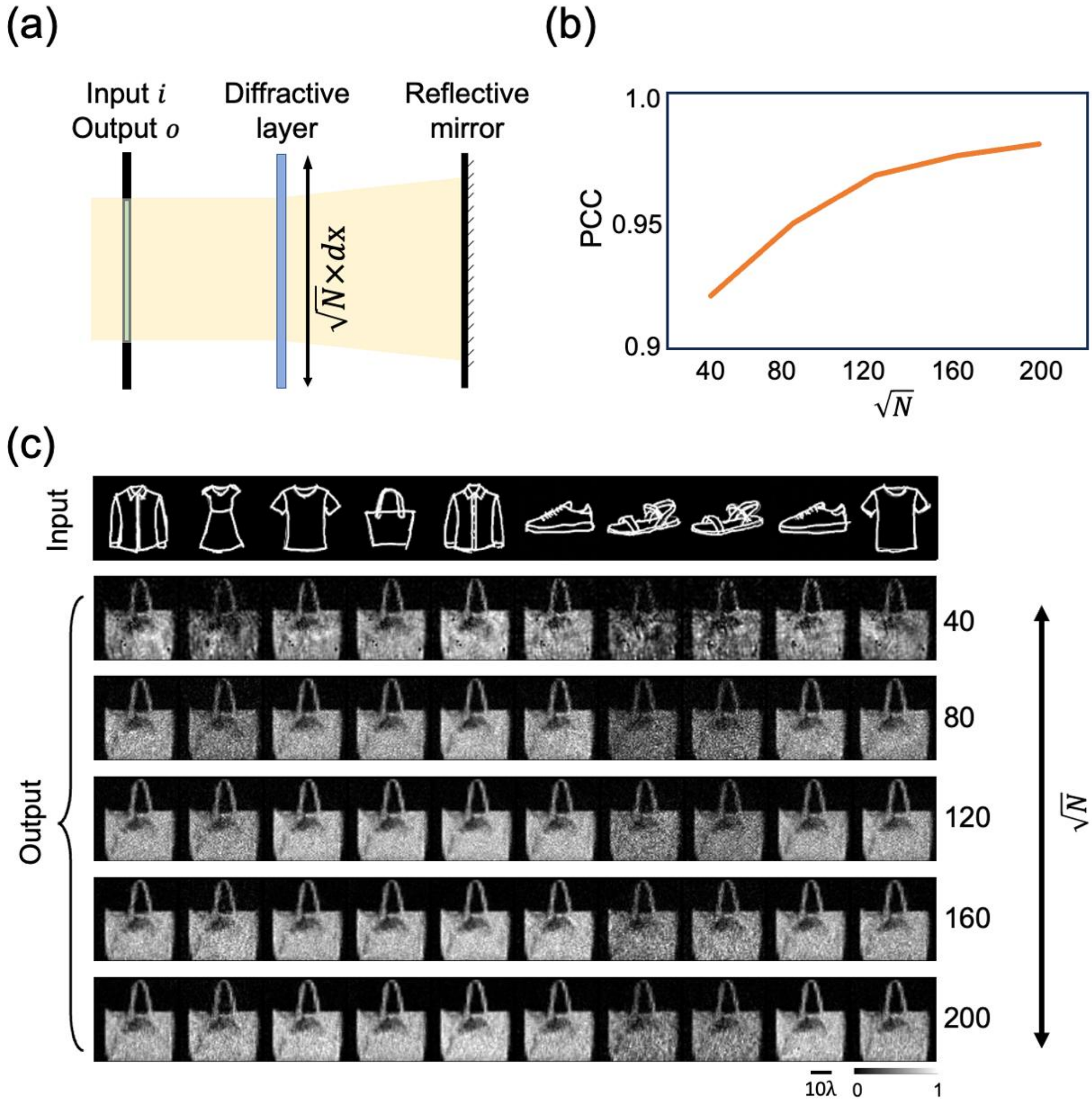

Figure S4. Evaluation of the performance of lying mirrors for varying number of diffractive features (N). (a) Schematic of a lying mirror with N diffractive features and a fixed pixel size (dx). (b) Quantitative performance analysis and (c) visualizations of the lying mirror outputs as a function of N. For publication, the original Fashion-MNIST input images have been replaced with representative images depicting the same object classes to comply with copyright requirements. These input images are shown for visualization only; the original images used in our figure/analysis correspond to image indices 9900–9909 in the test split and the original dataset order was preserved without shuffling. All reported experiments, quantitative results, and the corresponding output images were generated using the original benchmark datasets described in the Methods, and are unchanged.

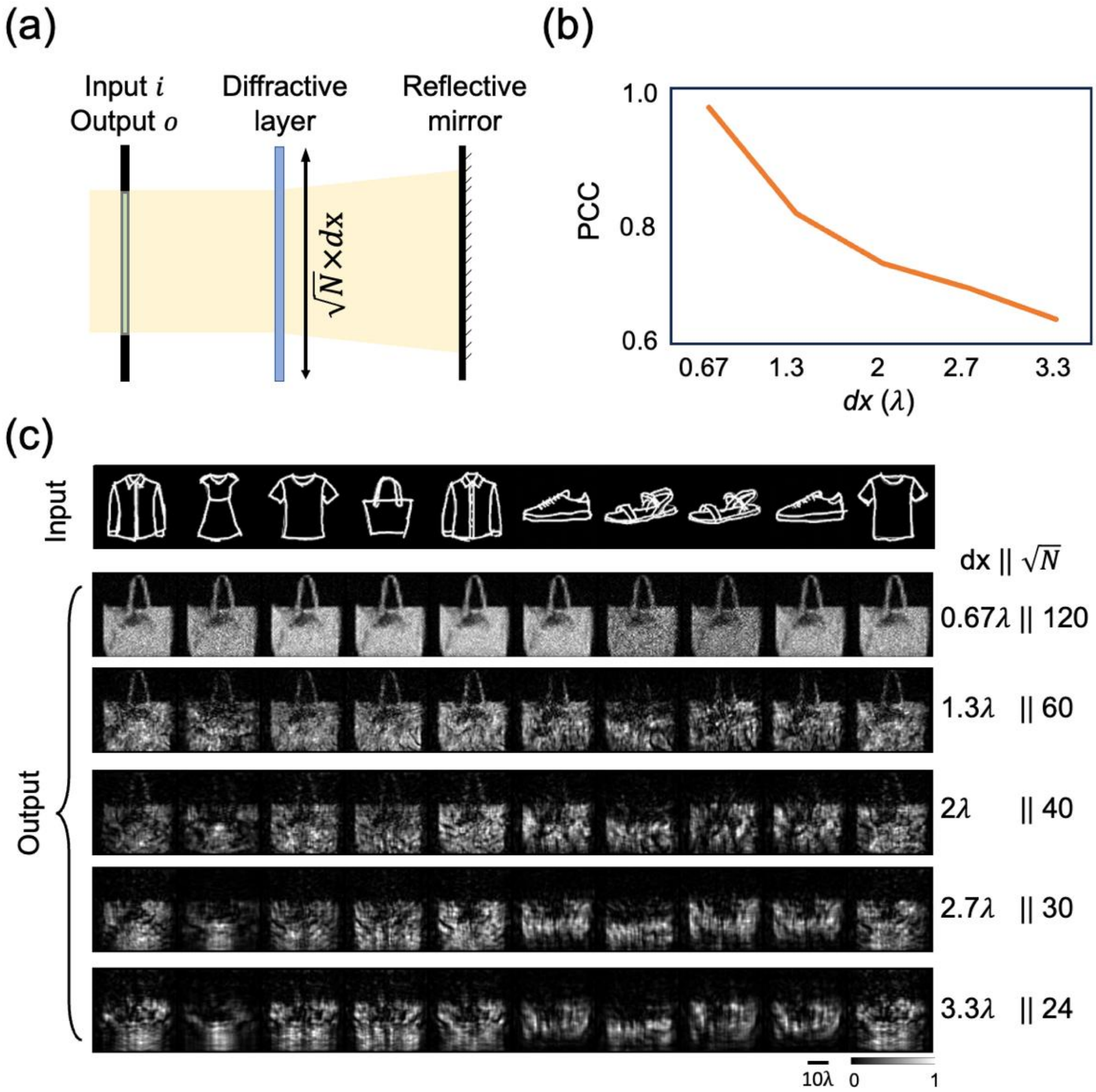

Figure S5. Evaluation of lying mirror performance for different pixel sizes (dx). (a) Schematic of a lying mirror with changing diffractive feature sizes (dx) while maintaining a fixed layer size ($\sqrt{N} \times d$x). (b) Quantitative performance analysis and (c) visualizations of the lying mirror outputs as a function of dx. For publication, the original Fashion-MNIST input images have been replaced with representative images depicting the same object classes to comply with copyright requirements. These input images are shown for visualization only; the original images used in our figure/analysis correspond to image indices 9900–9909 in the test split and the original dataset order was preserved without shuffling. All reported experiments, quantitative results, and the corresponding output images were generated using the original benchmark datasets described in the Methods, and are unchanged.

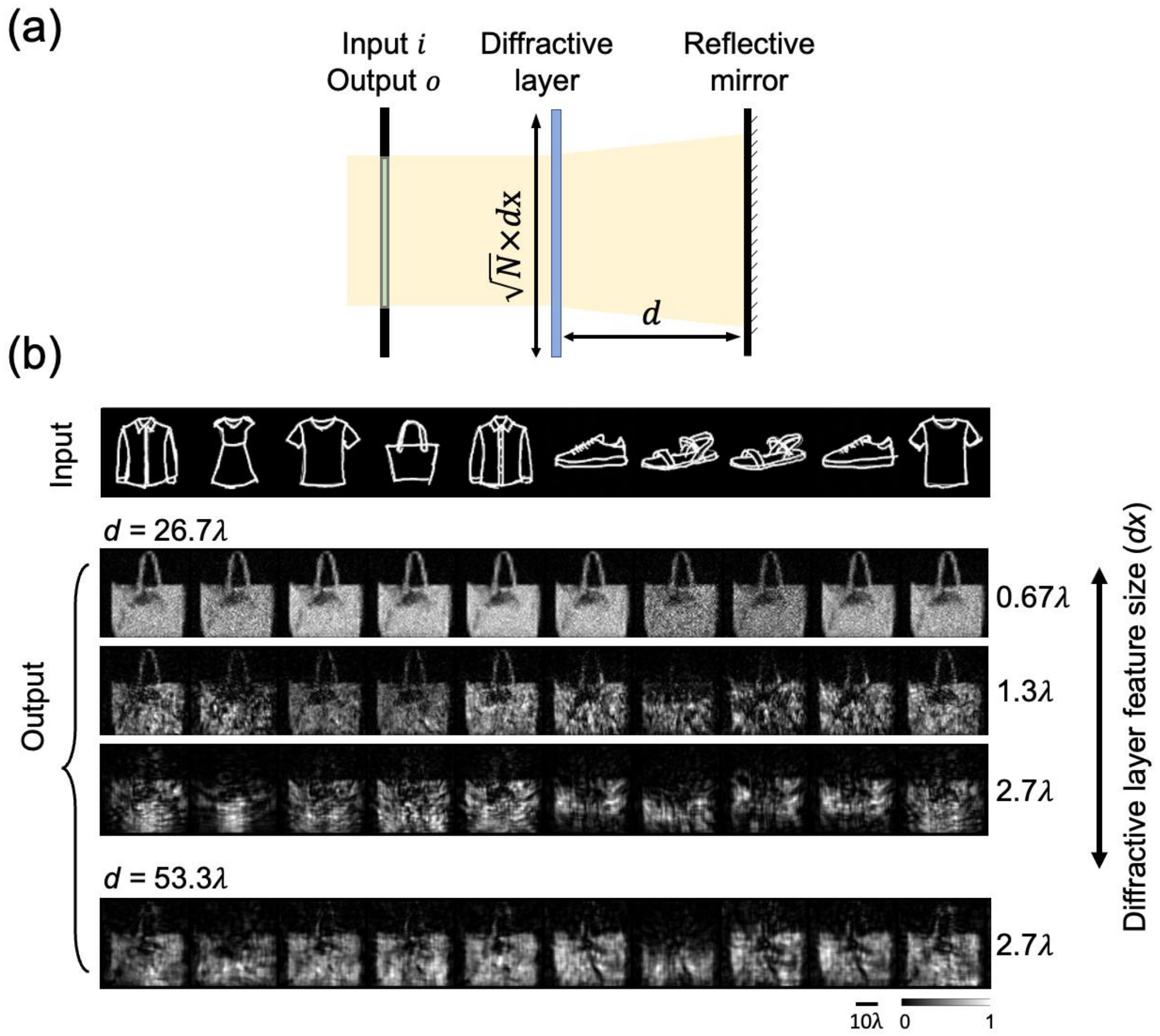

Figure S6. Evaluation of the performance of lying mirrors for different pixel sizes (dx) with a fixed total number of diffractive features (N). (a) Schematic of a lying mirror. (b) Visualizations of the lying mirror outputs as a function of dx. The results show that increasing the axial distance between the structured diffractive surface and the reflective mirror improves the lying mirror performance, even when larger pixel sizes are used. For publication, the original Fashion-MNIST input images have been replaced with representative images depicting the same object classes to comply with copyright requirements. These input images are shown for visualization only; the original images used in our figure/analysis correspond to image indices 9900–9909 in the test split and the original dataset order was preserved without shuffling. All reported experiments, quantitative results, and the corresponding output images were generated using the original benchmark datasets described in the Methods, and are unchanged.

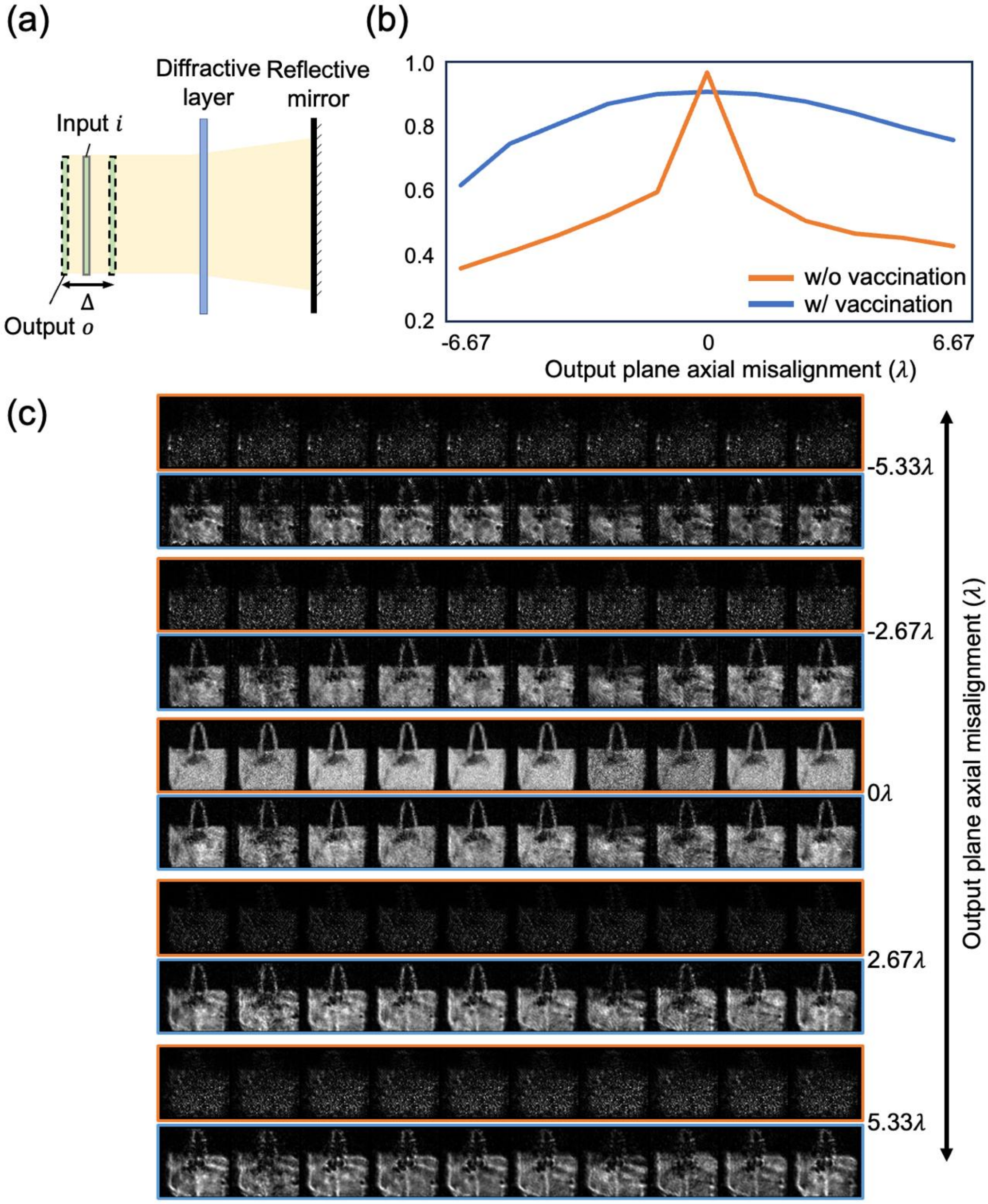

Figure S7. Evaluation of lying mirror performance under varying levels of output plane axial misalignments (Δ). (a) Schematic of a lying mirror with output plane axial misalignments (Δ). (b) Quantitative performance analysis and (c) image visualizations comparing the lying mirror outputs—with and without vaccination—across different axial misalignments (Δ). Orange: without vaccination; Blue: with vaccination.

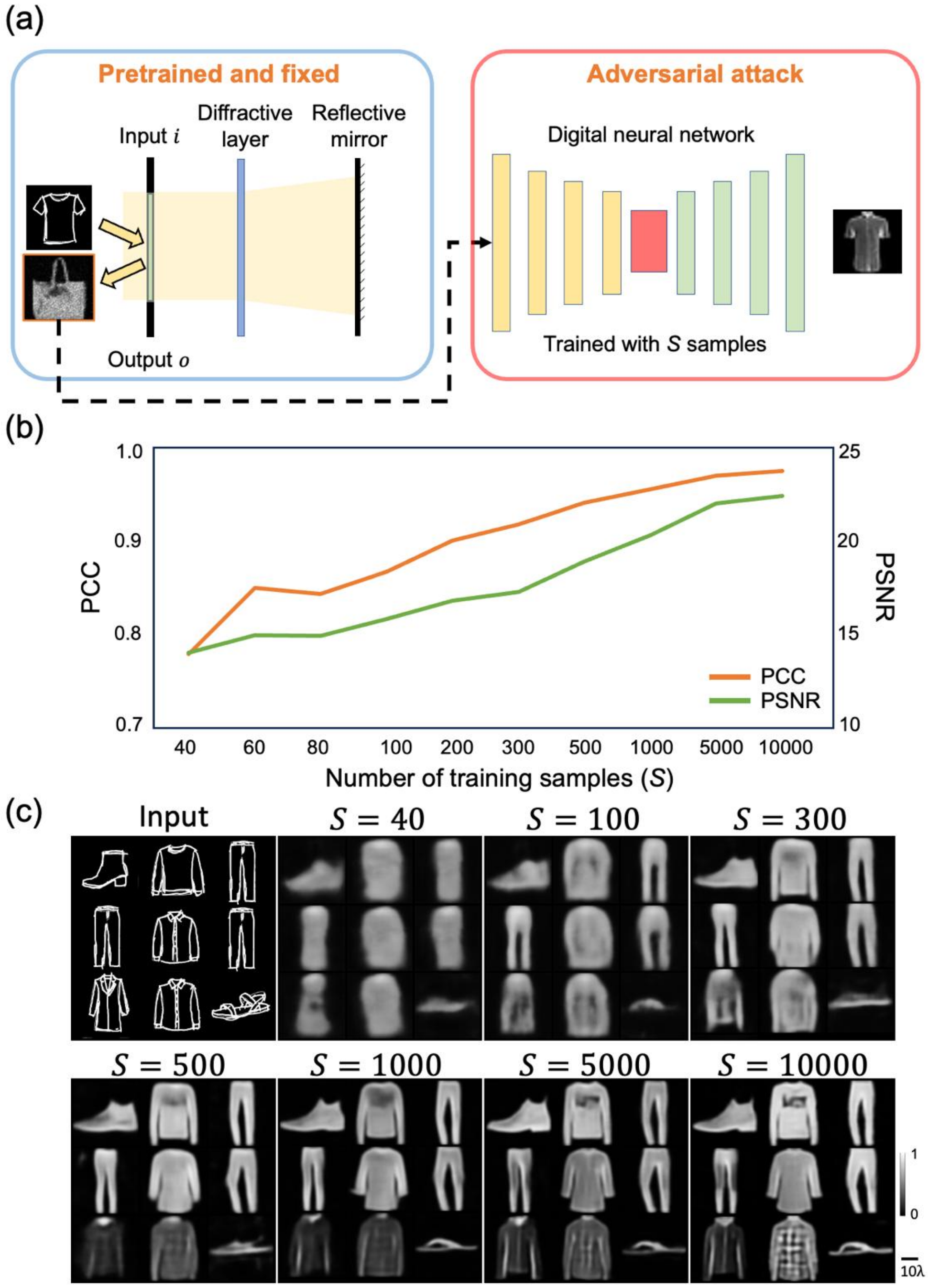

Figure S8. Adversarial attack on a lying mirror design using a digital neural network (backend). (a) Schematic illustrating the reconstruction of input images concealed by the lying mirror via a digital neural network. (b) Quantitative performance analysis and (c) image visualizations of reconstructions generated by the adversarial neural networks trained with different numbers (S) of training samples. For publication, the original Fashion-MNIST input images have been replaced with representative images depicting the same object classes to comply with copyright requirements. These input images are shown for visualization only; the original images used in our figure/analysis correspond to image indices 0–8 in the test split and the

original dataset order was preserved without shuffling. All reported experiments, quantitative results, and the corresponding output images were generated using the original benchmark datasets described in the Methods, and are unchanged.

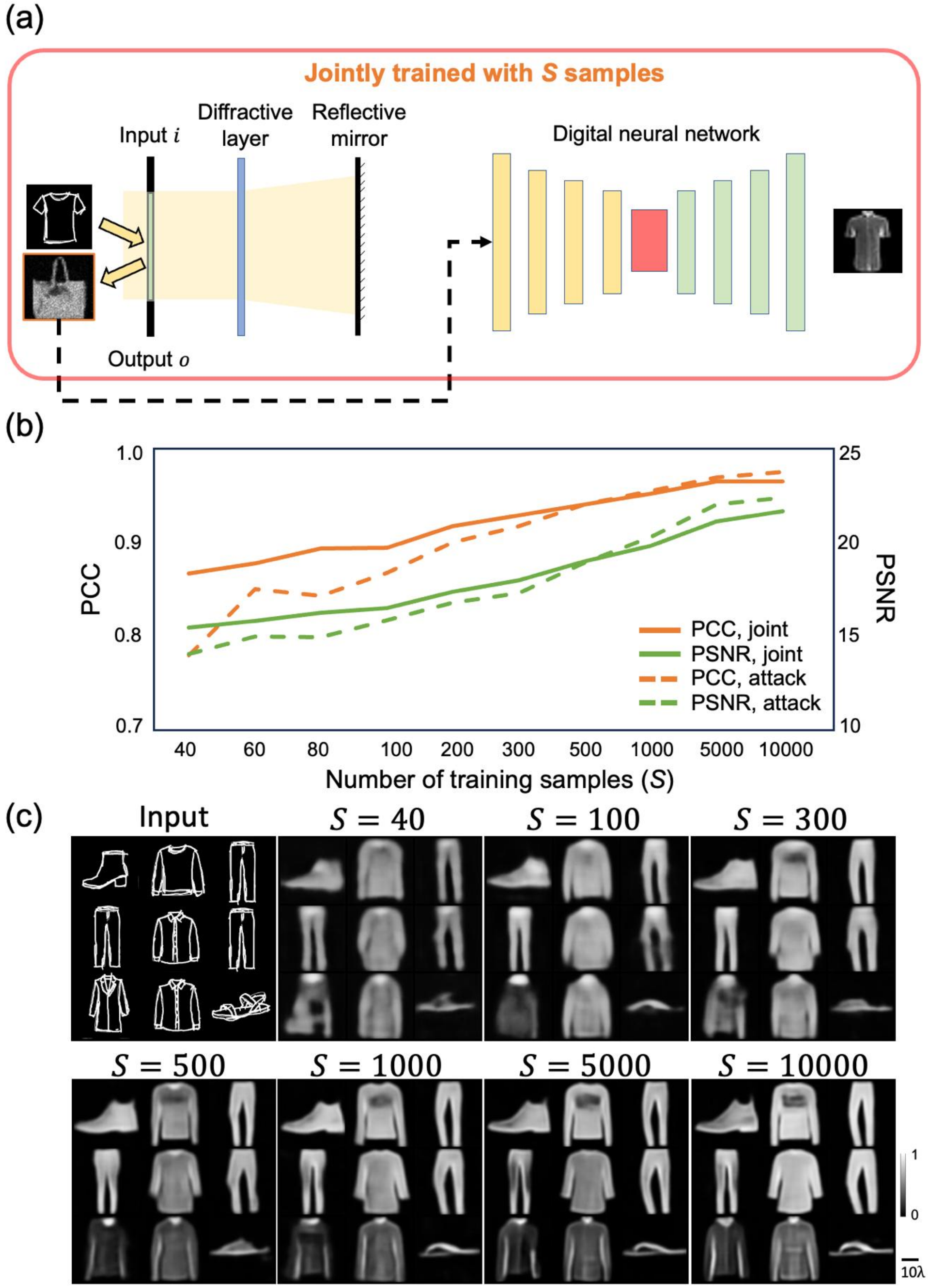

Figure S9. Joint training of a lying mirror with a digital neural network backend. (a) Schematic of the jointly trained system, which integrates a lying mirror for image hiding with a digital neural network for image reconstruction at the backend. (b) Quantitative performance analysis comparing the jointly trained

model and the adversarial attacks (demonstrated in Fig. S8) using PCC and PSNR metrics. (c) Visualizations of the image reconstructions from hybrid systems trained with different numbers (*S*) of training samples. For publication, the original Fashion-MNIST input images have been replaced with representative images depicting the same object classes to comply with copyright requirements. These input images are shown for visualization only; the original images used in our figure/analysis correspond to image indices 0–8 in the test split and the original dataset order was preserved without shuffling. All reported experiments, quantitative results, and the corresponding output images were generated using the original benchmark datasets described in the Methods, and are unchanged.

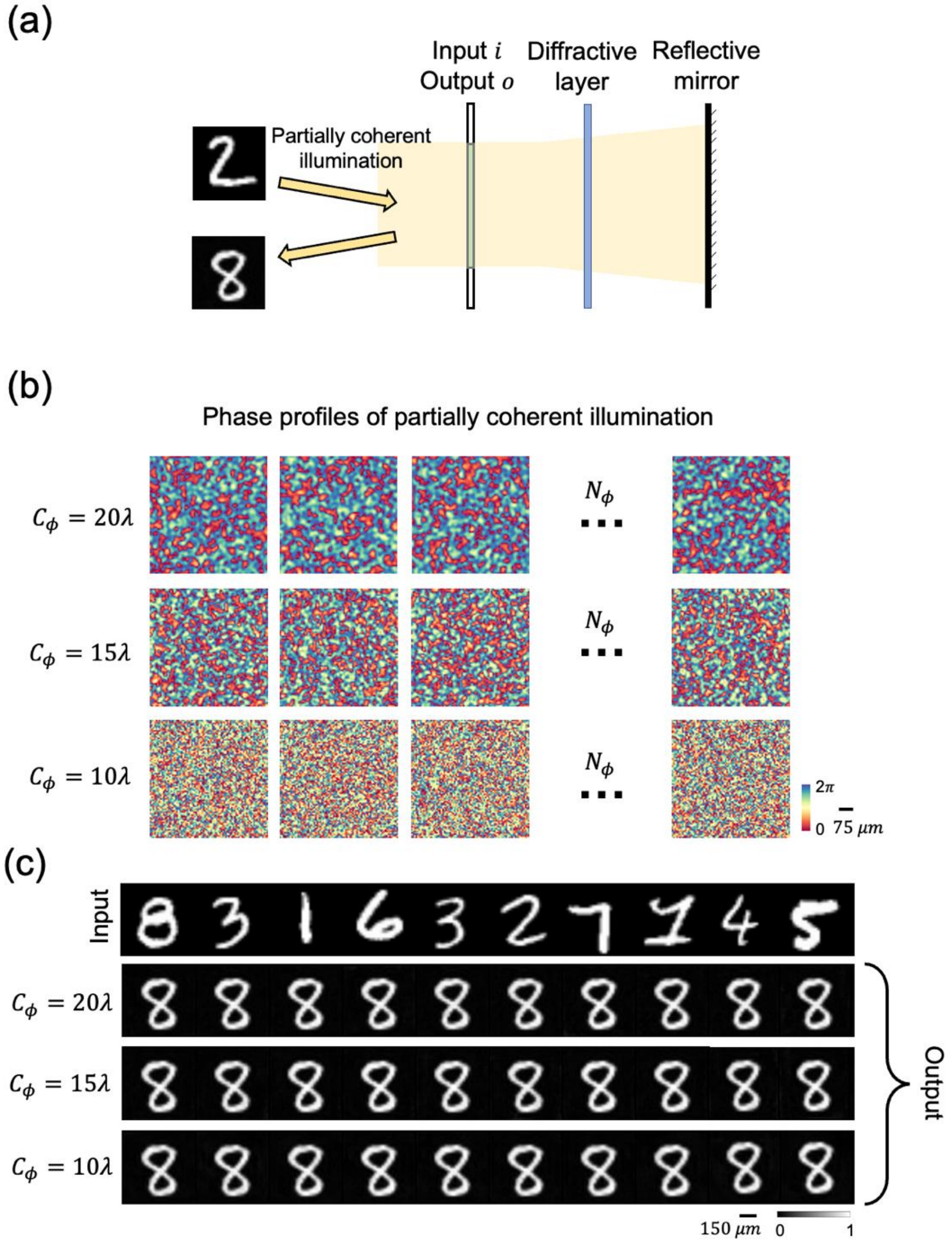

Figure S10. Lying mirrors with spatially partially coherent illumination. (a) Schematic illustration of a lying mirror under partially coherent illumination. (b) Three sets of random phase profiles, each set comprising N_ϕ phase distributions are illustrated. Every set corresponds to a distinct correlation length, C_ϕ, varying from 10λ to 20λ. (c) Visualizations of the lying mirror output images under partially coherent illumination light with different phase correlation lengths C_ϕ.

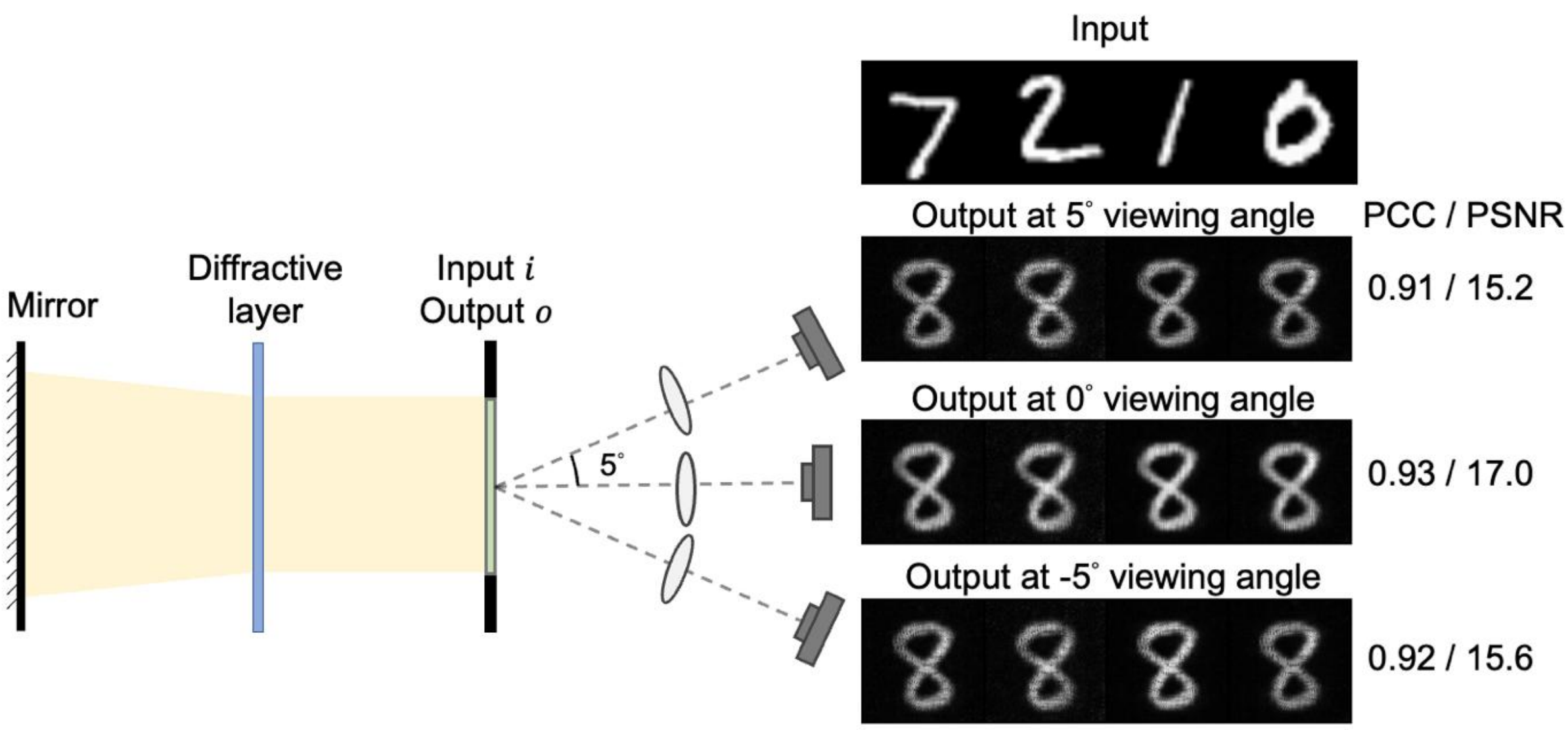

Figure S11. Deceptive outputs of a lying mirror viewed from different angles. This diffractive lying mirror was trained only with 0° viewing angle at its output, and its generalization was tested under ±5° viewing angles, which revealed a similar performance as the 0° viewing angle as indicated by the PCC and PSNR values at each viewing angle.
