# Supplementary material for: Lying mirror using structured surfaces
Source: Nat Commun. 2026 Aug 7;17:9140. doi: 10.1038/s41467-026-76488-2 (PMC13521926; doi:10.1038/s41467-026-76488-2)
Supplement: Supplementary file 2 [file 41467_2026_76488_MOESM2_ESM.pdf]

nature portfolio

Peer Review File

Lying mirror using structured surfaces

Corresponding Author: Dr Aydogan Ozcan

This file contains all reviewer reports in order by version, followed by all author rebuttals in order by version.

Version 0:

Reviewer comments:

Reviewer #1

(Remarks to the Author)
This manuscript introduces a "lying mirror" system, which is used to hide input information by transforming it into misleading, seemingly ordinary patterns. However, there are several issues with this manuscript:

1. As far as I know, the concept of using reflective surfaces to encode and deceive is not novel and has been widely reported in the literature. The novelty of this work is not clearly demonstrated.

2. The reverse process of the deception mirror is not shown in this work—is the pattern information recoverable and can it be effectively reversed? This is a critical issue that needs to be addressed to establish the practical utility of this system.

3. The potential use of this system in the military camouflage is mentioned but not clearly defined. If there are appropriate application scenarios, please highlight them.

4. The authors claim that the diffractive layer is optimized through deep learning, which could be a novel aspect of this manuscript. If this is the case, more details on the training and optimization process of the deep learning model are needed, such as the network architecture, data pre-processing for the training dataset, hyperparameter selection, and other relevant details that would allow for the replication of the results.

5. The term "all-optical" is used throughout the manuscript, but it is not clearly defined. It is suggested that the authors provide a clear definition of what they mean by "all-optical" in the context of this work.

6. The title of the manuscript is too broad and does not accurately reflect the specific content and contributions of this work. A more specific and descriptive title is needed to better capture the essence of this research.

Based on the concerns raised above, the current manuscript does not sufficiently support publication in Nature Communications and I recommend that the manuscript be rejected.

Reviewer #2

(Remarks to the Author)
The manuscript titled “Lying Mirror” by Yuhang Li et al. presents an intriguing exploration of optical neural network designs for manipulating images through light-matter interaction. The concept is both novel and accessible. The all-optical lying mirror has the capability to transform images at the speed of light, digitally switching to alternate camouflage images at high frame rates with minimal power consumption. Essentially, it rearranges incoming information into a desired format, which holds significant potential not only for imaging and display applications but also for advancements in reconfigurable intelligent surfaces and wireless communication. Overall, I find the manuscript to be engaging, well-written, and well-illustrated. Nevertheless, the study lacks an examination of the impact of incoherent light sources on image quality, which is directly applicable to most use cases and consequently limits the scope of this work. Additionally, the manuscript does not provide sufficient experimental details or explanations regarding the designs of the optical diffractive layers. Thus, I believe a major revision is necessary. Detailed comments and suggestions are outlined below.

1. The authors mention several potential applications for the lying mirror. However, for these applications, the light sources predominantly involve incoherent radiation and scattered light, which differs from the conditions demonstrated in the

manuscript. To enhance the credibility and impact of the authors' work, it is essential to present experimental evidence demonstrating the functionality of the diffractive optical processor with incoherent light. It would be valuable to understand how the degree of incoherent radiation, both spatially and temporally, impacts the quality of the camouflage images.

2. Please discuss the influence of pixel number and pixel size on the optical diffractive layer. A detailed description of the optimized design process is needed. In the section "Methods – Parameters and Digital Implementation for Numerical Analyses," it is unclear why the wavelength was set at a sub-millimeter scale instead of optical wavelengths, given this decision influences the diffractive design features and spacing between layers, differing by three orders of magnitude. Please provide clarification on this aspect.

3. All experimental results presented show successful lying images, but what about the unsuccessful attempts? It is crucial to include processes and discussions on how the authors identified optimized parameter sets (e.g., pixel number, pixel size) and which parameters significantly influence this work. Additionally, what occurs if the image plane deviates from the optimized position, and what is the tolerable distance?

4. While the Pearson Correlation Coefficient (PCC) serves as a good indicator for evaluating the structural similarity of images with a targeted image, it would be necessary to include other indicators, such as Mean Squared Error (MSE), to assess image quality more comprehensively. I suggest conducting a more thorough quantitative analysis of the lying mirror designs. In Figure 6, the PCC of random patterns with a uniform distribution appears to increase linearly as the ratio of active pixels rises. Could you provide an explanation for this observation? It seems that a PCC above 0.7 (Model 2) fails to transform into fake images. What would be the PCC threshold value to distinguish successful from unsuccessful cases?

Reviewer #3

(Remarks to the Author)
This manuscript describes an optical system that projects input image data into a deceiving pattern that differs from the input images. The optical system can be implemented either with a diffractive layer + a mirror, or a single diffractive layer on a reflective substrate, i.e., structured mirror. Three designs have been shown on three datasets, MNIST, Fashion-MNIST, and QuickDraw, to demonstrate the effectiveness of the method in simulation, and a real prototype is built for the structured mirror setup on MNIST dataset with three wavelengths for RGB channels. The authors also designed simulation experiments to demonstrate its robustness to noise, spatial shifts, rotation, and etc. The proposed technique might be useful for camouflage. The manuscript is well written and easy to follow.

Although the idea of lying mirror looks interesting, the setting is very limited -- in particular unlike a regular mirror it only works for one optimized viewing direction; consistent motion under observer parallax is not demonstrated. In such a setting a simple display would be a much more practical solution generate a deceiving image. In addition, there are some concerns about the implementation and technical details are not fully described in the current manuscript. We think the following questions need to be clarified and addressed before it can be published.

1. Choice of dummy output

Throughout the work, the demonstrated dummy output images seem to be one instance from the corresponding datasets. For example, "bag" for Fashion-MNIST, "8" for MNIST, and "panda" for QuickDraw. Essentially, such patterns still fall into the space of these datasets statistically. Then the problem being solved in this work is to find a diffractive layer that maps all the data entries into one point in the dataset's space. Although cross-dataset evaluation is performed in Fig. 3, these datasets do share similarities – simple binary or grayscale patterns. From a practical perspective, can one use arbitrary patterns that are not sampled from such datasets for the dummy output image? How does the system work if the dummy output is sampled from, say, ImageNet?

2. Deceiving or not-deceiving

Using an instance sampled from the mentioned datasets also raises another question about the effectiveness of deceiving the observer. Since the system is to "lie" about the original image, it does not do the job well if the input is "8" and the output is still "8", although they are different instances of "8" in the dataset (see Fig. 2). I.e. the approach works well in this case since the input and the distorted image are very close already, but that does not necessarily mean the method will work for more dissimilar parings. In particular, if the original image is out of distribution, there is no evidence that the output image is plausible.

3. Performance boundary evaluation analysis

In the manuscript, the authors analyzed the performance with respect to the sparsity of the input image. However, this is not done completely to help understand the performance boundary. Some simple extreme patterns could be done here. First, what is the output pattern for a point source? This corresponds to the lowest end for sparsity. Second, what is the output pattern for a uniform input, say all ones? Is it still true that "as the ratio of the active pixels within the input FOV increased, the output PCC values also increased"? Essentially, this is to extend the horizontal range in Fig. 6. If the statement holds true, does it simply mean that all-ones input would output the best dummy image? It is interesting to see if the optimized diffractive pattern is a CGH for the dummy output under uniform illumination.

4. Distance between the diffractive layer and mirror

From the diffractive + mirror design to the single structured mirror design, the difference seems to be the distance between the two components. The current manuscript does not talk much about how this distance impact the final performance. It would be helpful to carry out an experiment with different distances to analyze the performance to show that reducing this distance to 0 (the one used for the prototype) does not affect the performance if it is monotonic. However, it is also interesting to see if there exists an optimal distance that performs the best.

5. Training details

Some training details should be revealed, such as optimizer, learning rate, initialization of the diffractive patterns.

Reviewer #4

(Remarks to the Author)
I co-reviewed this manuscript with one of the reviewers who provided the listed reports. This is part of the Nature Communications initiative to facilitate training in peer review and to provide appropriate recognition for Early Career Researchers who co-review manuscripts.

Version 1:

Reviewer comments:

Reviewer #1

(Remarks to the Author)
I recommend rejecting the publication of this manuscript, since the novelty of this paper is insufficient to warrant publication in Nature Communications:

(1) The concept of a "lying mirror" that can disguise a wide variety of input images as specified target images has already been published in Bijie Bai et al., Information-hiding cameras: Optical concealment of object information into ordinary images. Sci. Adv. 10, eadn9420 (2024). DOI: 10.1126/sciadv.adn9420.

(2) The performance of the lying mirror, which operates effectively across multiple wavelengths and is insensitive to polarization changes, is determined by the intrinsic material properties and does not represent a special innovation.

Reviewer #2

(Remarks to the Author)
The authors have addressed all my comments. I recommend publishing it as it is.

Reviewer #3

(Remarks to the Author)
The latest revisions and new experiments have addressed some of the more technical concerns raised in the initial review, such as questions about generalization to different target images, observation conditions, etc.

What remains it the observation raised before about the fundamental limits of the technology that have no clear path for a solution. As written in the review:

"Although the idea of lying mirror looks interesting, the setting is very limited -- in particular unlike a regular mirror it only works for one optimized viewing direction; consistent motion under observer parallax is not demonstrated. In such a setting a simple display would be a much more practical solution generate a deceiving image."

Such fundamental limitations of the technology are not fairly discussed in the manuscript.

Reviewer #4

(Remarks to the Author)
I co-reviewed this manuscript with one of the reviewers who provided the listed reports. This is part of the Nature Communications initiative to facilitate training in peer review and to provide appropriate recognition for Early Career Researchers who co-review manuscripts.

Version 2:

Reviewer comments:

Reviewer #5

(Remarks to the Author)
I recommend acceptance of the re-revised manucsript.

Reviewer #6

(Remarks to the Author)

Open Access This Peer Review File is licensed under a Creative Commons Attribution 4.0 International License, which permits use, sharing, adaptation, distribution and reproduction in any medium or format, as long as you give appropriate credit to the original author(s) and the source, provide a link to the Creative Commons license, and indicate if changes were made.
In cases where reviewers are anonymous, credit should be given to 'Anonymous Referee' and the source.
The images or other third party material in this Peer Review File are included in the article's Creative Commons license, unless indicated otherwise in a credit line to the material. If material is not included in the article's Creative Commons license and your intended use is not permitted by statutory regulation or exceeds the permitted use, you will need to obtain permission directly from the copyright holder.
To view a copy of this license, visit https://creativecommons.org/licenses/by/4.0/

We sincerely thank the referees for their reviews and the constructive feedback that we have received on our manuscript "***Lying mirror***" submitted to *Nature Communications*.

As detailed below, we have revised our manuscript in response to the reviewers' comments. The original referee comments are shown in black color, whereas for ease of communication, our answers are provided in blue. Our revisions have also been marked in the main text and supplementary information files using yellow highlighting.

Summary of our Revisions:
We created a **Supplementary Information (SI) file** based on the reviewers' comments. As a quick summary, the following items have been revised and added to our manuscript's main text and SI, highlighted in yellow:

Revised sub-sections:

- **Changes to text:**
    - Title
    - Abstract
    - Introduction
    - Results
    - Discussion
    - Methods

New Figures Added:

- **Figure 9. Experimental demonstration of a structured lying mirror for broadband operation.**
- **Supplementary Fig. S1. Blind testing results of lying mirrors.**
- **Supplementary Fig. S2. Quantitative analysis of the performance of lying mirror outputs for different input images.**
- **Supplementary Fig. S3. Evaluation of the performance of lying mirrors under various axial distances between the diffractive layer and the standard reflective mirror.**
- **Supplementary Fig. S4. Evaluation of the performance of lying mirrors for varying number of diffractive features (N).**
- **Supplementary Fig. S5. Evaluation of lying mirror performance for different pixel sizes ($d\mathrm{x}$).**
- **Supplementary Fig. S6. Evaluation of the performance of lying mirrors for different pixel sizes ($d\mathrm{x}$) with a fixed total number of diffractive features (N).**
- **Supplementary Fig. S7. Evaluation of lying mirror performance under varying levels of output plane axial misalignments (Δ).**
- **Supplementary Fig. S8. Adversarial attack on a lying mirror design using a digital neural network (backend).**
- **Supplementary Fig. S9. Joint training of a lying mirror with a digital neural network backend.**
- **Supplementary Fig. S10. Lying mirrors with spatially partially coherent illumination.**

Reviewer #1:

This manuscript introduces a "lying mirror" system, which is used to hide input information by transforming it into misleading, seemingly ordinary patterns. However, there are several issues with this manuscript:

1. As far as I know, the concept of using reflective surfaces to encode and deceive is not novel and has been widely reported in the literature. The novelty of this work is not clearly demonstrated.

-- We thank the reviewer for raising this important point, which gives us an opportunity to further elaborate on the novelty of our work. While we acknowledge that the use of reflective surfaces for encoding and deception has been explored in previous literature, our work distinguishes itself with several unique advancements. First, the system's working principle is not restricted to a few specific objects, unlike previous works; it can camouflage a wide variety of input images, including those significantly different from the training data, into designated target images (see e.g., **Fig. 3**). This adaptability further distinguishes it from conventional deception techniques that typically rely on fixed image sets covering limited/restricted examples or predefined surface structures. Furthermore, As demonstrated in our experimental results (**Figs. 8** and **9**), the lying mirror operates effectively across several wavelengths, making it adaptable to varying lighting conditions and enhancing its applicability for broadband operation. Furthermore, our design consists of an isotropic diffractive layer and a reflective mirror, making it inherently insensitive to polarization changes. The isotropic nature of the diffractive layer ensures stable performance regardless of the polarization state of the incoming light.

Accordingly, we have highlighted these points in the **Discussion section** of the revised manuscript to better emphasize the novelty and some of the major contributions of our work:

> *"…The lying mirror offers a new approach to optical deception, distinguishing itself from conventional reflective surfaces through its broadband operation, adaptability to various input images, and polarization insensitivity. As demonstrated in our experiments (Figs. 8 and 9), the system functions effectively across multiple wavelengths, making it resilient to varying lighting conditions. Additionally, although trained on specific image datasets, it generalizes well to input images significantly different from the training data, camouflaging them into designated target images (Fig. 3). This adaptability contrasts with conventional techniques that typically require predefined input-output mappings, limiting their flexibility."*

> *"…The lying mirror framework consists of an isotropic diffractive layer and a reflective mirror, which are polarization-insensitive. These features make the lying mirror a promising candidate for applications in camouflage, imaging, and optical information processing, offering a robust and adaptable solution for various desired optical transformations."*

2. The reverse process of the deception mirror is not shown in this work—is the pattern information recoverable and can it be effectively reversed? This is a critical issue that needs to be addressed to establish the practical utility of this system.

-- We thank the reviewer for raising the discussion about whether the encrypted patterns can be effectively recovered. Following the referee's suggestion, we conducted an **adversarial attack** on the lying mirror design by employing a digital neural network trained on a set of paired input-output images, as shown in **Supplementary Fig. S8**. We evaluated the recovery quality using networks trained with varying numbers of training samples. Our results indicate that with a limited number of training samples,

the digital neural network cannot recover finer details of the objects (see **Supplementary Fig. S8c**). As the number of training samples increases, the reconstruction improves markedly: with 500 paired training samples, finer features become discernible, and with up to 10,000 training samples, higher-quality reconstructions, including logos and intricate patterns, were achieved; see **Supplementary Fig. S8c**.

Furthermore, we explored a jointly trained system in which the lying mirror and the back-end digital neural network were optimized together, as illustrated in **Supplementary Fig. S9**. Compared to the adversarial attack, the jointly trained system better captures the complex relationships inherent in the data, leading to more robust and precise reconstructions of the input objects.

We included these points within the **Discussion section** and **Methods section** of the revised manuscript:

> *"…We also conducted an adversarial attack on the lying mirror design by employing a digital neural network trained on a set of paired input-output images (see **Supplementary Fig. S8**). Our evaluations involved adversarial attacks using neural networks trained on varying sample sizes, which revealed that a limited training set could not recover the finer details of the input objects (**Supplementary Fig. S8c**). Notably, with 500 training examples (i.e., paired input-output images), finer features began to emerge, and with ~10,000 training samples, the adversarial network produced high-quality reconstructions of the input images, capturing intricate details. Additionally, we investigated a jointly trained system in which both the lying mirror and the digital neural network were optimized simultaneously (**Supplementary Fig. S9**). This integrated approach enables the system to capture complex data relationships, resulting in more robust and precise reconstructions of the input objects compared to the adversarial attacks demonstrated in **Supplementary Fig. S8**."*

> *"…The adversarial digital neural networks that were employed to reconstruct/transform the dummy images back into the input images, reported in **Supplementary Figs. S8 and S9**, were based on the U-Net architecture[32]."*

3. The potential use of this system in the military camouflage is mentioned but not clearly defined. If there are appropriate application scenarios, please highlight them.

-- This is indeed an important point. The lying mirror may have potential applications in optical deception, particularly in scenarios involving coherent laser-based reconnaissance technologies (e.g., LiDAR or structured illumination methods). If integrated into viewing windows or periscopes, it could influence the observed output, potentially making it more difficult for adversaries to identify assets accurately. Additionally, the system might be explored for situations where coherent illumination is employed for detection. Specifically, it could be considered for integration into semi-transparent materials used in military contexts, such as stealth fabrics or light-filtering windows in vehicles. In these cases, it may assist in modulating light, potentially altering how objects are perceived through adversarial optical devices and affecting detection outcomes.

We have revised the **Introduction section** of the manuscript to include the above discussed points:

> *"…Furthermore, lying mirrors, which manipulate visual perception through optical distortions, may also present applications in defense and security, including camouflage and deception[5–9]. For example, these mirrors could contribute to enhancing camouflage by altering the appearance of equipment, vehicles, or personnel under specific viewing conditions, making them appear differently shaped, smaller, or larger, thus confusing adversaries and delaying target identification[10–14]. In structured optical environments such as viewing ports, surveillance*

windows, or underwater detection systems, the technology may also help disrupt adversarial imaging techniques, reducing the effectiveness of target identification… In scenarios where coherent illumination is used for reconnaissance, lying mirrors might be integrated into semi-transparent materials or optical interfaces to alter the perceived appearance of objects, potentially making them seem differently shaped or visually obscured."

4. The authors claim that the diffractive layer is optimized through deep learning, which could be a novel aspect of this manuscript. If this is the case, more details on the training and optimization process of the deep learning model are needed, such as the network architecture, data pre-processing for the training dataset, hyperparameter selection, and other relevant details that would allow for the replication of the results.

-- We thank the reviewer for suggesting that we provide more details on the training process. To clarify, the optimization process does not rely on a traditional neural network architecture. **Instead, we employed a deep learning-based optimization approach to design the lying mirror, where the trainable parameters are the phase modulation values of the diffractive layers**. These layers are treated as learnable components that manipulate the input light field to achieve the desired optical transformation. This training process involves feeding input images through the optical system, calculating the resulting output images, and comparing them with predefined target output images. The loss was computed using a PCC-based loss function, as detailed in the **Methods section**, which maximized the structural similarity between the output and the target. Using automatic differentiation, gradients of the loss function with respect to the phase modulation values of the diffractive layers were backpropagated. These gradients were then used to iteratively update the phase values via gradient descent, ensuring convergence to the desired optical transformation. The above process is very similar to the standard training procedure used in deep learning frameworks. For training, we used standard datasets, including MNIST, Fashion-MNIST, and QuickDraw, to simulate the input images. Further details about hyperparameters, such as batch size, learning rate and training epoch, are provided in the "Parameters and Digital Implementation for Numerical Analyses" subsection of the **Methods section**.

The above points have been added to the **Results section** and **Methods section** of the revised manuscript:

*"…We employed a deep learning-based optimization approach, which involved feeding the input image dataset through the lying mirror, calculating the corresponding outputs, and comparing them to the predefined target output images (see the **Methods** section). "*

"…, where $\phi(x, y)$ represents the phase modulation value for the corresponding diffractive feature, which forms the trainable parameters of the lying mirror."

"…For the broadband experimental results and the corresponding lying mirror design, multiple illumination wavelengths were randomly sampled from $520\ nm$ to $570\ nm$ in each batch. Each wavelength followed the corresponding forward model described above.

With the above numerical forward model, we employed a deep learning-based approach to optimize the phase values of the diffractive layers. The training process involved feeding the input images through the optical system, calculating the output, and comparing it with the predefined target "dummy" output image to compute the loss. Then, by using automatic differentiation, we backpropagated the gradients to the trainable parameters (i.e., the phase modulation values of the diffractive layer). Leveraging a gradient descent-based optimization method, commonly used in training deep neural networks, we iteratively optimized the lying mirror design to ensure that the output images closely matched the predefined target images."

*"…The adversarial digital neural networks that were employed to reconstruct/transform the dummy images back into the input images, reported in **Supplementary Figs. S8 and S9**, were based on the U-Net architecture[32].*

Multi-wavelength, broadband, and spatially partially coherent lying mirror models were trained with a batch size of 16 for 30 epochs, while all the other models were trained with a batch size of 150 for 200 epochs. The Adam optimizer was used with default parameters and a learning rate of 0.001. The layers were initialized with zero phase. The training process used PyTorch (v1.11.0, Meta Platforms Inc.) on a GeForce RTX 3090 GPU (NVIDIA Inc.)."

5. The term "all-optical" is used throughout the manuscript, but it is not clearly defined. It is suggested that the authors provide a clear definition of what they mean by "all-optical" in the context of this work.

-- In our work, "all-optical" refers to the fact that the optical transformation performed by the lying mirror from input to output is entirely performed in the optical domain, solely relying on the propagation and programmed diffraction of light. **This means that no electronic or digital components are involved in the actual image transformation process, and once the diffractive layer is fabricated, the system would operate passively without requiring any external power**. We have included these points in the **Introduction section** of the revised manuscript:

"…This transformation process, from input to output images, is purely performed in the optical domain through the propagation and programmed diffraction of light. As a result, no electronic or digital processing is required, and once the diffractive layer is fabricated, the system operates without any external power consumption."

6. The title of the manuscript is too broad and does not accurately reflect the specific content and contributions of this work. A more specific and descriptive title is needed to better capture the essence of this research.

-- We sincerely thank the reviewer for their suggestion to refine the title for greater specificity. Accordingly, we revised the title to "***Lying mirror using structured surfaces***".

Reviewer #2:

The manuscript titled "Lying Mirror" by Yuhang Li et al. presents an intriguing exploration of optical neural network designs for manipulating images through light-matter interaction. The concept is both novel and accessible. The all-optical lying mirror has the capability to transform images at the speed of light, digitally switching to alternate camouflage images at high frame rates with minimal power consumption. Essentially, it rearranges incoming information into a desired format, which holds significant potential not only for imaging and display applications but also for advancements in reconfigurable intelligent surfaces and wireless communication. Overall, I find the manuscript to be engaging, well-written, and well-illustrated. Nevertheless, the study lacks an examination of the impact of incoherent light sources on image quality, which is directly applicable to most use cases and consequently limits the scope of this work. Additionally, the manuscript does not provide sufficient experimental details or explanations regarding the designs of the optical diffractive layers. Thus, I believe a major revision is necessary. Detailed comments and suggestions are outlined below.

-- We sincerely thank the reviewer for their thoughtful evaluation and constructive feedback on our manuscript, which helped us further enhance the quality of the manuscript.

1. The authors mention several potential applications for the lying mirror. However, for these applications, the light sources predominantly involve incoherent radiation and scattered light, which differs from the conditions demonstrated in the manuscript. To enhance the credibility and impact of the authors' work, it is essential to present experimental evidence demonstrating the functionality of the diffractive optical processor with incoherent light. It would be valuable to understand how the degree of incoherent radiation, both spatially and temporally, impacts the quality of the camouflage images.

-- While our initial experimental demonstrations focused on coherent and multi-wavelength illumination, we have also investigated the performance of the lying mirror under **spatially partially coherent illumination**. These new analyses and numerical simulations reported in ***Supplementary Fig. S10*** provide evidence that the lying mirror can effectively function even when spatial coherence is reduced down to a phase correlation length of a few wavelengths.

Additionally, our existing experimental results involve multi-wavelength illumination. To further extend our analysis, we have trained and experimentally tested a new broadband lying mirror capable of operating across a wide range of wavelengths (500nm – 600nm – see the ***new Figure 9***). This broadband design and its experimental validation demonstrate the robustness of the lying mirror under various lighting conditions.

We have included these results and analyses within the **Abstract, Results, Discussion** and **Methods section** of the revised manuscript, quoted below:

> "…Furthermore, we created a broadband lying mirror that operates across a continuous spectral range, enhancing its adaptability under diverse illumination conditions."

> *"…Beyond the RGB lying mirror design demonstrated above, we further designed a lying mirror for broadband operation, trained across a continuum spanning $520nm - 570nm$ range (see the **Methods** section for training details). The schematic of the experimental setup is shown in **Fig. 9a**, where two SLMs were used as the input plane and the lying mirror, respectively. We evaluated the performance of this design across various wavelengths selected from $500nm$ to $600nm$, extending beyond the design/training spectral range. As shown in **Fig. 9b**, the results indicate that the lying mirror achieved high-quality concealment of input images within its training spectral range. Outside this region, the output images still maintained PCC values above 0.85 over a spectrum of $500 - 600\ nm$, demonstrating generalization to input wavelengths that remain outside of the training spectral range (see **Fig. 9b**). Additionally, we experimentally tested this design under different wavelength illuminations using 10 randomly selected MNIST images, as shown in **Fig. 9c**. All the input images were successfully transformed into the target dummy handwritten digit '8', experimentally validating the lying mirror's ability to operate across a continuum spanning $500 - 600\ nm$; see **Fig. 9c**."*

> *"…As shown in **Supplementary Fig. S10**, three lying mirrors were trained under partially coherent light with different phase correlation lengths (see the **Methods** section for details), all of which successfully concealed the input images by transforming them into a predefined ordinary image."*

> *"*
>
> ***Simulation of Spatially Partially Coherent Illumination***
>
> *To model the propagation of a partially coherent field, we define the phase profile $\phi_i(x,y)$ of the incident field as[30]:*
>
> $$\phi_i(x,y) = mod\left[\frac{2\pi}{\lambda}W(x,y) * G(x,y), 2\pi\right] \quad (11),$$

where '' denotes the 2D convolution operation. Here, $W(x, y)$ follows a normal distribution and $G(x, y)$ represents a Gaussian smoothing kernel. We numerically tailored these phase profiles $\phi_i(x, y)$ to achieve the desired correlation lengths C_ϕ, which were determined using the autocorrelation function $R_\phi(x, y)$:*

$$R_\phi(x, y) = \mathcal{F}^{-1}\{|\mathcal{F}\{\varphi_i(x, y)\}|^2\} = exp\left(-\frac{\pi(x^2+y^2)}{C_\phi}\right) \quad (12).$$

With Eq. (12), we numerically estimated C_ϕ based on 4096 randomly generated phase profiles $\phi_i(x, y)$, given specific $W(x, y)$ and $G(x, y)$.

The time-average intensity $\hat{O}$ of the lying mirror under spatially partially coherent illumination was computed as[26–28,31]:

$$\hat{O} = \langle \widehat{O_i} \rangle = \lim_{N_\phi \to \infty} \frac{1}{N_\phi} \sum_{i=1}^{N_\phi} \widehat{O_i} \quad (13).$$

During the training of partially coherent lying mirror designs, we used $N_\phi^{train} = 16$ random phase profiles per input image, while the final performance evaluations employed $N_\phi^{test} = 4096$ random phase profiles for each test object/image."

"In the experimental demonstration shown in Fig. 9, the structured lying mirror was trained to transform its input images into a target image under broadband illumination ranging from $520\ nm$ to $570\ nm$. Following the above-outlined procedures, we trained the structured mirror to process phase-only input images while capturing intensity at the output plane. During the training, each original 28×28 grayscale MNIST image was linearly upscaled to 112×112 pixels (with a pixel size of $8\ \mu m$) using nearest-neighbor interpolation. The structured mirror consisted of 300×300 trainable diffractive features, each with a lateral size of $8\ \mu m$. The axial distance between the input and the structured mirror was set to $120.1\ mm$, while the distance from the structured mirror to the CMOS image sensor was $96.4\ mm$. The central 112×112 pixels within the output FOV were cropped for the loss function calculation.

For the simulation of a lying mirror under spatially partially coherent illumination, the wavelength was selected as $532\ nm$. During the training, each original 28×28 grayscale MNIST image was linearly upscaled to 80×80 pixels (with a pixel size of $8\ \mu m$) using nearest-neighbor interpolation. The structured mirror consisted of 200×200 trainable diffractive features, each with a lateral size of $8\ \mu m$. The axial distances were the same as those used in the broadband lying mirror designs. The central 80×80 pixels within the output FOV were cropped to calculate the time-average intensity $\hat{O}$ for the loss function calculation. All quantitative data analyses, including PCC values, PSNR values and loss function calculations, were performed on raw data without any image contrast enhancement applied."

"…For the broadband lying mirror experiments, the same illumination source was used as before; we also used two SLMs for the object plane and the structured lying mirror plane (Meadowlark XY Phase Series, pixel pitch: $8\mu m$, resolution: 1920×1200; HOLOEYE PLUTO-2.1, pixel pitch $8\mu m$, resolution 1920×1080). The axial distance between the input object plane (SLM) to the structured mirror plane was $120.1\ mm$, and the distance between the structured mirror to the sensor plane was $96.4\ mm$."

2. Please discuss the influence of pixel number and pixel size on the optical diffractive layer. A detailed description of the optimized design process is needed. In the section "Methods – Parameters and Digital Implementation for Numerical Analyses," it is unclear why the wavelength was set at a sub-millimeter scale instead of optical wavelengths, given this decision influences the diffractive design features and spacing between layers, differing by three orders of magnitude. Please provide clarification on this aspect.

-- We thank the reviewer for the thoughtful comments and the opportunity to clarify these aspects; we have performed new analyses on these points and created new **Supplementary Figs. S4, S5 and S6**. The number of diffractive features and pixel size are critical parameters in the design of the optical diffractive layer, as they directly influence the performance of the system. The total number of diffractive features impacts the degrees of freedom available for the optical transformation, where a larger pixel count improves the fidelity of the output but requires greater computational resources during training. As shown in **Supplementary Fig. S4,** when we increased the number of diffractive features while keeping the pixel size constant, the output performance improved.

Similarly, larger pixel sizes result in a lower spatial resolution, which can reduce the system's ability to transform the finer features of the input images. In contrast, smaller pixel sizes enable finer control of the light field but may pose challenges during fabrication. As shown in **Supplementary Fig. S5,** the output performance degrades as the pixel size increases while keeping the overall layer size unchanged. Nonetheless, this performance loss due to a large pixel size can be mitigated by increasing the axial distance between the structured diffractive surface and the reflective mirror. As shown in **Supplementary Fig. S6**, with a greater axial distance, the performance of the lying mirror with a large diffractive feature size can be comparable to that of a system with smaller pixels.

These performance trade-offs have been carefully considered in our design, and a discussion of these factors has been added to the **Discussion section** of the revised manuscript.

> *"…The design of the optical diffractive layer involves critical trade-offs between the number of diffractive features and pixel size. Increasing the total number of diffractive features enhances the degrees of freedom available for performing a desired optical transformation and improves the output image fidelity, as shown in **Supplementary Fig. S4**. As for the pixel size, smaller pixels, approaching ~λ/2, offer finer control over the light field and achieve better performance, as shown in **Supplementary Figs. S5-S6**; however, such smaller diffractive features can also pose challenges in fabrication and optimization. Larger pixels can reduce the capability of the lying mirror to conceal the input information effectively, as shown in **Supplementary Fig. S6**. Nonetheless, this performance loss due to a large pixel size can be mitigated by increasing the axial distance between the structured diffractive surface and the reflective mirror, as demonstrated in **Supplementary Fig. S6**."*

Regarding the choice of illumination wavelength: the underlying principles and design framework behind lying mirrors can be adapted to operate at different parts of the spectrum, including the visible band, by accordingly scaling the diffractive features with respect to the wavelength; **stated differently, without the need to redesign the diffractive features, one can scale the phase features of each layer with respect to the operational wavelength**. Our experimental results at the visible spectrum (**Figs. 8 and 9, which covered 480-600 nm**) demonstrate the feasibility and success of this approach.

Following the referee's suggestions, we have updated the "Parameters and Digital Implementation for Numerical Analyses" sub-section of the **Methods section** to include these details:

> *"…For the lying mirror designs operating under monochromatic coherent illumination, a numerical analysis was conducted at a wavelength of λ = 0.75 mm. While these analyses were performed in the terahertz range, the lying mirror design is inherently scalable with respective to the illumination wavelength λ; it can be adapted to operate in different spectral ranges, including the visible part of the spectrum, by simply scaling its diffractive features according to the illumination wavelength. This scalability was demonstrated in our experimental results, which showcased the successful implementation of the lying mirror designs in the visible spectrum."*

3. All experimental results presented show successful lying images, but what about the unsuccessful attempts? It is crucial to include processes and discussions on how the authors identified optimized parameter sets (e.g., pixel number, pixel size) and which parameters significantly influence this work.

Additionally, what occurs if the image plane deviates from the optimized position, and what is the tolerable distance?

-- In our analyses, we found that the number of diffractive features and pixel size are critical parameters that directly influence system performance. As discussed in our earlier response above, the total number of diffractive features impacts the degrees of freedom available for the optical transformation, where a larger pixel count improves the fidelity of the output but requires greater computational resources during training. As shown in **Supplementary Fig. S4,** when we increased the number of diffractive features while keeping the pixel size constant, the output performance improved. Similarly, larger pixel sizes result in a lower spatial resolution, which can reduce the system's ability to transform the finer features of the input images. In contrast, smaller pixel sizes enable finer control of the light field but may pose challenges during fabrication. As shown in **Supplementary Fig. S5,** the output performance degrades as the pixel size increases while keeping the overall layer size unchanged. Nonetheless, this performance loss due to a large pixel size can be mitigated by increasing the axial distance between the structured diffractive surface and the reflective mirror. As shown in **Supplementary Fig. S6**, with a greater axial distance, the performance of the lying mirror with a large diffractive feature size can be comparable to that of a system with smaller pixels.

These performance trade-offs have been carefully considered in our designs, and a discussion of these factors has been added to the **Discussion section** of the revised manuscript, as quoted earlier in our responses.

Furthermore, we investigated the impact of random deviations from the optimal output plane position. As shown in the newly added **Supplementary Fig. S7**, the performance declines when the image plane shifts from its optimal axial location. To mitigate this, we introduced a "vaccination" process during training, deliberately incorporating image plane position shifts. Consequently, the trained lying mirror maintains robust performance (PCC > 0.6) over a wide range of shifts.

Accordingly, we included these points in the **Results section** of the revised manuscript:

> *"…The design of the optical diffractive layer involves critical trade-offs between the number of diffractive features and pixel size. Increasing the total number of diffractive features enhances the degrees of freedom available for performing a desired optical transformation and improves the output image fidelity, as shown in **Supplementary Fig. S4**. As for the pixel size, smaller pixels, approaching ~λ/2, offer finer control over the light field and achieve better performance, as shown in **Supplementary Figs. S5-S6**; however, such smaller diffractive features can also pose challenges in fabrication and optimization. Larger pixels can reduce the capability of the lying mirror to conceal the input information effectively, as shown in **Supplementary Fig. S6**. Nonetheless, this performance loss due to a large pixel size can be mitigated by increasing the axial distance between the structured diffractive surface and the reflective mirror, as demonstrated in **Supplementary Fig. S6**. Additionally, our lying mirror exhibits sensitivity to the output plane's axial position, with the output image quality degrading with deviations from the optimal position (see **Supplementary Fig. S7**). To enhance the robustness of a lying mirror against such misalignments, we introduced a "vaccination" strategy during the training[25], deliberately incorporating image plane position shifts. As demonstrated in **Supplementary Fig. S7**, this vaccination approach allows the system to maintain its performance with a PCC of > 0.6 over a wide range of axial misalignments, ensuring reliable operation even when the image plane deviates from its ideal position. These findings underscore the importance of balancing design parameters to achieve optimal and robust system performance."*

4. While the Pearson Correlation Coefficient (PCC) serves as a good indicator for evaluating the structural similarity of images with a targeted image, it would be necessary to include other indicators, such as Mean Squared Error (MSE), to assess image quality more comprehensively. I suggest

conducting a more thorough quantitative analysis of the lying mirror designs. In Figure 6, the PCC of random patterns with a uniform distribution appears to increase linearly as the ratio of active pixels rises. Could you provide an explanation for this observation? It seems that a PCC above 0.7 (Model 2) fails to transform into fake images. What would be the PCC threshold value to distinguish successful from unsuccessful cases?

-- The Pearson Correlation Coefficient (PCC) quantifies the linear correlation between two images, making it a suitable metric for our analysis. In addition to PCC, we introduced the Peak Signal-to-Noise Ratio (PSNR) as a metric that reflects the Mean Squared Error (MSE), with the results presented in the revised **Fig. 6** and **Supplementary Fig. S2**. The PSNR exhibits a similar trend to the PCC, further supporting our analysis. We would also like to clarify that in the **original Fig. 6**, the images shown within the Model 2 results were samples of the input noise pattern rather than the output images. Accordingly, we have added additional visualizations in the revised **Fig. 6** to clarify our results better. Based on these results, the threshold for distinguishing a successful case from an unsuccessful one is around 0.5. Regarding the observation that the PCC of random patterns increases with the ratio of active pixels, we believe that each active point in the input plane contributes to forming the target dummy image.

Following the referee's suggestions, we have included these points in the **Results** and **Methods** section of the revised manuscript:

> *"…This trend suggests that a higher count of active pixels generally enhances the model's ability to conceal input images. We also employed the Peak Signal-to-Noise Ratio (PSNR) metric to assess the output image quality (see the* ***Methods*** *section), with a similar performance observed in* ***Supplementary Fig. S2****. Additionally, tests under special illumination conditions—point source and uniform illumination—further confirmed the same trend. Figure 6b visualizes the input and output images for Model 3, with the corresponding PCC and PSNR values also indicated in each case."*

> *"…We also used the PSNR metric to assess the output image quality, which was defined as:*
>
> $$PSNR = 10\,log_{10}\left(\frac{MAX_{I_{target}}^2}{MSE(O, I_{target})}\right) \quad (10),$$
>
> *where $MAX_{I_{target}}^2$ is the maximum possible pixel value of the target "dummy" image (equal to 1), and $MSE(O, I_{target})$ is the Mean Square Error between the output and target images."*

Reviewer #3:

This manuscript describes an optical system that projects input image data into a deceiving pattern that differs from the input images. The optical system can be implemented either with a diffractive layer + a mirror, or a single diffractive layer on a reflective substrate, i.e., structured mirror. Three designs have been shown on three datasets, MNIST, Fashion-MNIST, and QuickDraw, to demonstrate the effectiveness of the method in simulation, and a real prototype is built for the structured mirror setup on MNIST dataset with three wavelengths for RGB channels. The authors also designed simulation experiments to demonstrate its robustness to noise, spatial shifts, rotation, and etc. The proposed technique might be useful for camouflage. The manuscript is well written and easy to follow.

Although the idea of lying mirror looks interesting, the setting is very limited -- in particular unlike a regular mirror it only works for one optimized viewing direction; consistent motion under observer parallax is not demonstrated. In such a setting a simple display would be a much more practical solution

generate a deceiving image. In addition, there are some concerns about the implementation and technical details are not fully described in the current manuscript. We think the following questions need to be clarified and addressed before it can be published.

-- We sincerely thank the referee for the insightful comments and constructive feedback, which helped us to enhance the quality and clarity of the manuscript.

1. Choice of dummy output

Throughout the work, the demonstrated dummy output images seem to be one instance from the corresponding datasets. For example, "bag" for Fashion-MNIST, "8" for MNIST, and "panda" for QuickDraw. Essentially, such patterns still fall into the space of these datasets statistically. Then the problem being solved in this work is to find a diffractive layer that maps all the data entries into one point in the dataset's space. Although cross-dataset evaluation is performed in Fig. 3, these datasets do share similarities – simple binary or grayscale patterns. From a practical perspective, can one use arbitrary patterns that are not sampled from such datasets for the dummy output image? How does the system work if the dummy output is sampled from, say, ImageNet?

-- We thank the reviewer for the insightful comment. Following the referee's suggestion, we have used the images from ImageNet as the dummy output, the results of which are shown in the newly added **Supplementary Fig. S1**. To accommodate the increased complexity of the ImageNet output, we employed a larger diffractive layer, which enabled us to achieve better performance. Our results demonstrate that all the input images can be successfully transformed into the targeted output images, highlighting the capability of our lying mirror to generate more complex images.

These results have been included in the **Results section** of the revised manuscript:

> *"…We also trained three additional lying mirror designs that transformed the Fashion-MNIST, MNIST, and QuickDraw datasets into a target output image selected from ImageNet using a larger diffractive layer. These results, shown in **Supplementary Fig. S1**, demonstrate the capability of our lying mirror framework to handle more intricate image transformations."*

2. Deceiving or not-deceiving

Using an instance sampled from the mentioned datasets also raises another question about the effectiveness of deceiving the observer. Since the system is to "lie" about the original image, it does not do the job well if the input is "8" and the output is still "8", although they are different instances of "8" in the dataset (see Fig. 2). I.e. the approach works well in this case since the input and the distorted image are very close already, but that does not necessarily mean the method will work for more dissimilar parings. In particular, if the original image is out of distribution, there is no evidence that the output image is plausible.

-- We would like to clarify that the purpose of **Fig. 2** was to demonstrate the system's internal generalization capabilities within the same dataset, where the training and testing images share statistical similarities. It shows that the lying mirror could consistently map various inputs from a given dataset (e.g., different "8" instances in MNIST) to a predefined dummy output image (e.g., a specific "8" from the same dataset). This analysis emphasizes the ability of the lying mirror to generalize across variations within the dataset rather than overfitting to specific input-output pairings.

Figure 3 evaluates cross-dataset performance, where the lying mirror was tested with input images from datasets that were **not** used during training (Fashion-MNIST and QuickDraw). While these datasets may share some statistical similarities, this evaluation provides initial evidence of the system's capacity to handle out-of-training-distribution inputs. **To further assess the generalization**

capabilities of our lying mirror framework, we conducted additional analyses using input images from ImageNet, which are more complex and diverse compared to the images used currently. As shown in the revised **Fig. 3**, all three lying mirror designs successfully transformed natural images into their corresponding predefined dummy outputs (e.g., "8," "bag," or "panda"). These results demonstrate the system's external generalization ability to process inputs that are out-of-distribution compared to the training dataset, offering promising potential for practical applications.

Accordingly, we have included the above points in the **Results section** of the revised manuscript:

> *"…In addition to these internal generalization tests, where the lying mirrors were tested with randomly selected new/unseen objects from the same image dataset used during training, we also assessed the external generalization capability of the same lying mirrors using image datasets different from the training data distribution. For example, Model 1, initially trained with the Fashion-MNIST dataset, was blindly tested using the MNIST, QuickDraw and ImageNet datasets. These successful external generalization tests, visualized in **Fig. 3**, indicate that our lying mirrors can consistently camouflage various randomly selected input images into the designated target images, despite the fact that these inputs are significantly different from the training image data and never encountered during training or design phase of the lying mirror. The average PCC values for Model 1 tested on MNIST, QuickDraw and ImageNet images were 0.84, 0.85 and 0.92, respectively. It is worth noting that the ImageNet dataset images are more diverse and distinct compared to those from the other two datasets, highlighting the lying mirror's external generalization ability to process inputs that are out-of-distribution compared to the training dataset. Although there was some performance degradation compared to the blind testing results with internal generalization data, the lying mirror still successfully produced deceptive images matching the desired dummy image structure at the output FOV. Similar analyses were also applied to Model 2, trained with MNIST, and Model 3, trained with QuickDraw, revealing the successful external generalization of these lying mirror designs. Each model effectively converted respective inputs into uniform target images, validating the adaptability and effectiveness of our lying mirror designs. We also trained three additional lying mirror designs that transformed the Fashion-MNIST, MNIST, and QuickDraw datasets into a target output image selected from ImageNet using a larger diffractive layer. These results, shown in **Supplementary Fig. S1**, demonstrate the capability of our lying mirror framework to handle more intricate image transformations.."*

3. Performance boundary evaluation analysis
In the manuscript, the authors analyzed the performance with respect to the sparsity of the input image. However, this is not done completely to help understand the performance boundary. Some simple extreme patterns could be done here. First, what is the output pattern for a point source? This corresponds to the lowest end for sparsity. Second, what is the output pattern for a uniform input, say all ones? Is it still true that "as the ratio of the active pixels within the input FOV increased, the output PCC values also increased"? Essentially, this is to extend the horizontal range in Fig. 6. If the statement holds true, does it simply mean that all-ones input would output the best dummy image? It is interesting to see if the optimized diffractive pattern is a CGH for the dummy output under uniform illumination.

-- We thank the reviewer for raising this interesting discussion. Following the referee's suggestions, we conducted additional analyses using a point source and a uniform input (all-ones). The results are consistent with our observation that "*a higher count of active pixels generally enhances the model's ability to conceal input images,*" with the all-ones input yielding the best dummy image (PCC = 0.98). However, it is also worth noting that the lying mirror is capable of producing similarly high-quality images with structured input patterns that require a much smaller ratio of active pixels, as shown in **Fig. 6a**. This further validates the importance of structured input patterns, as the lying mirror learns the spatial distribution of information during the training to effectively perform its concealing function.

Accordingly, we have revised **Fig. 6** and included the above points in the **Results section**:

> *"…To further investigate other factors influencing the image transformation performance of lying mirrors, we quantified the ratio of the active pixels—defined as the proportion of the pixels within the input FOV that exceed a specific intensity threshold (empirically set as 0.1). In addition to three datasets used earlier, i.e., MNIST, Fashion MNIST and QuickDraw, we also evaluated the lying mirror model's performance using binary random input patterns and random patterns with a uniform distribution U[0,1] – these constitute external test datasets never used during the training of our lying mirrors. Across all three lying mirror models, we observed that as the ratio of the active pixels within the input FOV increased, the output PCC values also increased, irrespective of the dataset used for training (**Fig. 6a**). This trend suggests that a higher count of active pixels generally enhances the model's ability to conceal input images. We also employed the Peak Signal-to-Noise Ratio (PSNR) metric to assess the output image quality (see the Methods section), with a similar performance observed in **Supplementary Fig. S2**. Additionally, tests under special illumination conditions—point source and uniform illumination—further confirmed the same trend. **Figure 6b** visualizes the input and output images for Model 3, with the corresponding PCC and PSNR values also indicated in each case.."*

4. Distance between the diffractive layer and mirror

From the diffractive + mirror design to the single structured mirror design, the difference seems to be the distance between the two components. The current manuscript does not talk much about how this distance impact the final performance. It would be helpful to carry out an experiment with different distances to analyze the performance to show that reducing this distance to 0 (the one used for the prototype) does not affect the performance if it is monotonic. However, it is also interesting to see if there exists an optimal distance that performs the best.

-- We thank the reviewer for raising this interesting discussion. In response, we conducted additional analyses across a range of axial distances between the diffractive element and the mirror. Our results indicate that increasing the distance leads to improved performance, with longer distances yielding better output quality. These findings have been incorporated into the revised manuscript, and the newly added **Supplementary Fig. S3** provides a more comprehensive analysis of the system's behavior. We included these points within the **Results section** of the revised manuscript:

> *"…Additional results for various axial distances between the optimized diffractive layer and the standard reflective mirror are provided in **Supplementary Fig. S3**, which shows that performance increases as the axial distance is increased."*

5. Training details

Some training details should be revealed, such as optimizer, learning rate, initialization of the diffractive patterns.

-- Following the referee's suggestion, we have included more training details in the **Methods section**:

> *"…Multi-wavelength, broadband, and spatially partially coherent lying mirror models were trained with a batch size of 16 for 30 epochs, while all the other models were trained with a batch size of 150 for 200 epochs. The Adam optimizer was used with default parameters and a learning rate of 0.001. The layers were initialized with zero phase. The training process used PyTorch (v1.11.0, Meta Platforms Inc.) on a GeForce RTX 3090 GPU (NVIDIA Inc.)."*

Reviewer #4:

I co-reviewed this manuscript with one of the reviewers who provided the listed reports. This is part of the Nature Communications initiative to facilitate training in peer review and to provide appropriate recognition for Early Career Researchers who co-review manuscripts.

-- We sincerely thank the reviewer for co-reviewing this manuscript. We greatly value the thoughtful suggestions provided, which have been instrumental in improving the quality of our work.

We sincerely thank the referees for their comments in the last two rounds of reviews and the constructive feedback that we have received on our manuscript "***Lying mirror using structured surfaces***" submitted to *Nature Communications*.

In light of the new comments received from Reviewers 1 and 3, we have revised our manuscript to address these additional concerns.

As detailed below, we have revised our manuscript in response to the reviewers' comments. The original referee comments are shown in black color, whereas for ease of communication, our answers are provided in blue. Our revisions have also been marked in the main text and supplementary information files using yellow highlighting.

Summary of our Revisions:
As a quick summary, the following items have been revised and added to our manuscript's main text and SI, highlighted in yellow:

Revised sub-sections:

- **Changes to text:**
  - Results
  - Discussion

New Figures Added:

- **Supplementary Fig. S11. Deceptive outputs of a lying mirror viewed from different angles.** This diffractive lying mirror was trained only with 0° viewing angle at its output, and its generalization was tested under ±5° viewing angles, which revealed a similar performance as the 0° viewing angle as indicated by the PSNR and PCC values at each viewing angle.

Reviewer #1:

I recommend rejecting the publication of this manuscript, since the novelty of this paper is insufficient to warrant publication in Nature Communications:
(1) The concept of a "lying mirror" that can disguise a wide variety of input images as specified target images has already been published in Bijie Bai et al., Information-hiding cameras: Optical concealment of object information into ordinary images. Sci. Adv. 10, eadn9420 (2024). DOI: 10.1126/sciadv.adn9420.
-- We appreciate the reviewer's reference to Bai et al. (Sci. Adv. 10, eadn9420, 2024) – our former publication. However, we would like to respectfully clarify several important points. First, we'd like to note that this concern was not raised during the initial review, nor was the cited paper mentioned at that time, thus precluding our opportunity to address it previously. This represents a new point introduced beyond the 6 technical points that we comprehensively responded to in the earlier round (for clarity of communication, our former Response Letter, summarizing our responses to the 6 technical questions that Reviewer 1 raised, is attached at the end of this document).

The system presented by Bai et al. significantly differs from this work in terms of system architecture, operational domain, methods and goals. **Their approach formulates a camera that is**

in transmission-mode and non-reconfigurable, demonstrated within the terahertz regime, where wavelengths are approximately 1000 times longer than the visible spectrum. In contrast, our manuscript introduces a reconfigurable reflective mirror system designed explicitly for visible wavelengths, realized through a programmable structured mirror. **Moreover, whereas Bai et al.'s system uses static 3D-fabricated components, our method employs spatial light modulators to achieve reconfigurability. Additionally, the reflective nature of our system facilitates compact integration and enhanced compatibility with mirror-based optical platforms, clearly distinguishing it from Bai et al.'s design and goals**.

(2) The performance of the lying mirror, which operates effectively across multiple wavelengths and is insensitive to polarization changes, is determined by the intrinsic material properties and does not represent a special innovation.
-- We respectfully disagree with this interpretation. **Our system achieves multi-wavelength operation explicitly through diffractive optimization, not through reliance on intrinsic material properties such as refractive index modulation or material dispersion engineering**. Our design strategy utilizes a thin structured surface coupled with free-space propagation, optimized explicitly for multiple illumination wavelengths simultaneously. **Importantly, lying mirrors can even be designed under flat dispersion, i.e., assuming the same refractive index across wavelengths**. This highlights that our broadband behavior arises from the computational design of the diffractive pattern, not from any wavelength-dependent material response. **This mechanism is fundamentally different from that of metamaterials or engineered structures that require precise tuning of refractive indices or anisotropic material responses to achieve spectral or polarization control**.

To make these points clearer, we note that in our current experimental results (**Figs. 8-9**), we used a flat dispersion model, where the refractive index was treated as constant across wavelengths. The phase delay variation was attributed solely to wavelength scaling, not material dispersion. The successful multi-wavelength and broadband performance that was experimentally observed under this assumption directly confirms that the system's functionality arises from the learned diffractive design through our deep learning-based optimization strategies, rather than any intrinsic material properties.

Accordingly, we have revised the **Results section** of the manuscript to include these points:

> *"…It is worth noting that in these experimental designs, we used a flat dispersion model, i.e., the refractive index was assumed to be constant across all wavelengths. As a result, the phase delays were solely governed by the wavelength-dependent phase kernel, rather than material dispersion-related effects. The successful experimental demonstrations of both multi-wavelength and broadband performance of lying mirrors provide direct evidence that the functionality of the lying mirrors arises from the learned diffractive surfaces enabled by our deep learning-based optimization strategy, rather than from any intrinsic material properties."*

We trust these clarifications effectively address the concerns and reaffirm the originality and significance of our contributions. In addition to these revisions outlined above, we also attached at the end of this document our former Response Letter, for completeness, summarizing our responses to the 6 technical questions that Reviewer 1 raised in the earlier round of the reviews.

Reviewer #2:
The authors have addressed all my comments. I recommend publishing it as it is.

-- We thank the reviewer for their time and effort in evaluating our work.

Reviewer #3:
The latest revisions and new experiments have addressed some of the more technical concerns raised in the initial review, such as questions about generalization to different target images, observation conditions, etc.

What remains it the observation raised before about the fundamental limits of the technology that have no clear path for a solution. As written in the review:
"Although the idea of lying mirror looks interesting, the setting is very limited -- in particular unlike a regular mirror it only works for one optimized viewing direction; consistent motion under observer parallax is not demonstrated. In such a setting a simple display would be a much more practical solution generate a deceiving image."

Such fundamental limitations of the technology are not fairly discussed in the manuscript.
-- We sincerely thank the reviewer for acknowledging that the latest revisions and new experiments have addressed the technical concerns raised in the initial review.

Regarding the remaining concern about the viewing direction, we would like to clarify the context in which this comment first appeared in their report. **In the initial review round, we thoroughly addressed five specific questions raised by Reviewer 3**. The above-quoted statement regarding parallax and viewing direction also appeared in the initial review of the referee but it was phrased as a general observation – not as a question to be addressed. **See below the quote from the earlier report of Referee 3, also attached here at the end of this document**:

> *"Although the idea of lying mirror looks interesting, the setting is very limited -- in particular unlike a regular mirror it only works for one optimized viewing direction; consistent motion under observer parallax is not demonstrated. In such a setting a simple display would be a much more practical solution to generate a deceiving image. In addition, there are some concerns about the implementation and technical details are not fully described in the current manuscript.*
>
> ***We think the following questions need to be clarified and addressed before it can be published..."***

In summary, the referee's earlier report clearly stated, "***We think the following questions need to be clarified and addressed before it can be published***..." **and there was no mention of the viewing direction in these following 5 questions that the referee specifically asked to be addressed, which we thoroughly did in our former Response Letter, also attached at the end of this document**.

In regard to the viewing angle: as demonstrated by our new simulation results shown below (**included in our newly added Supplementary Fig. S11**), the same deceptive images (as desired) were observed in our "Lying Mirror" output when viewed from different angles (±5°), confirming that our system maintains its functionality across multiple viewing directions; see below:

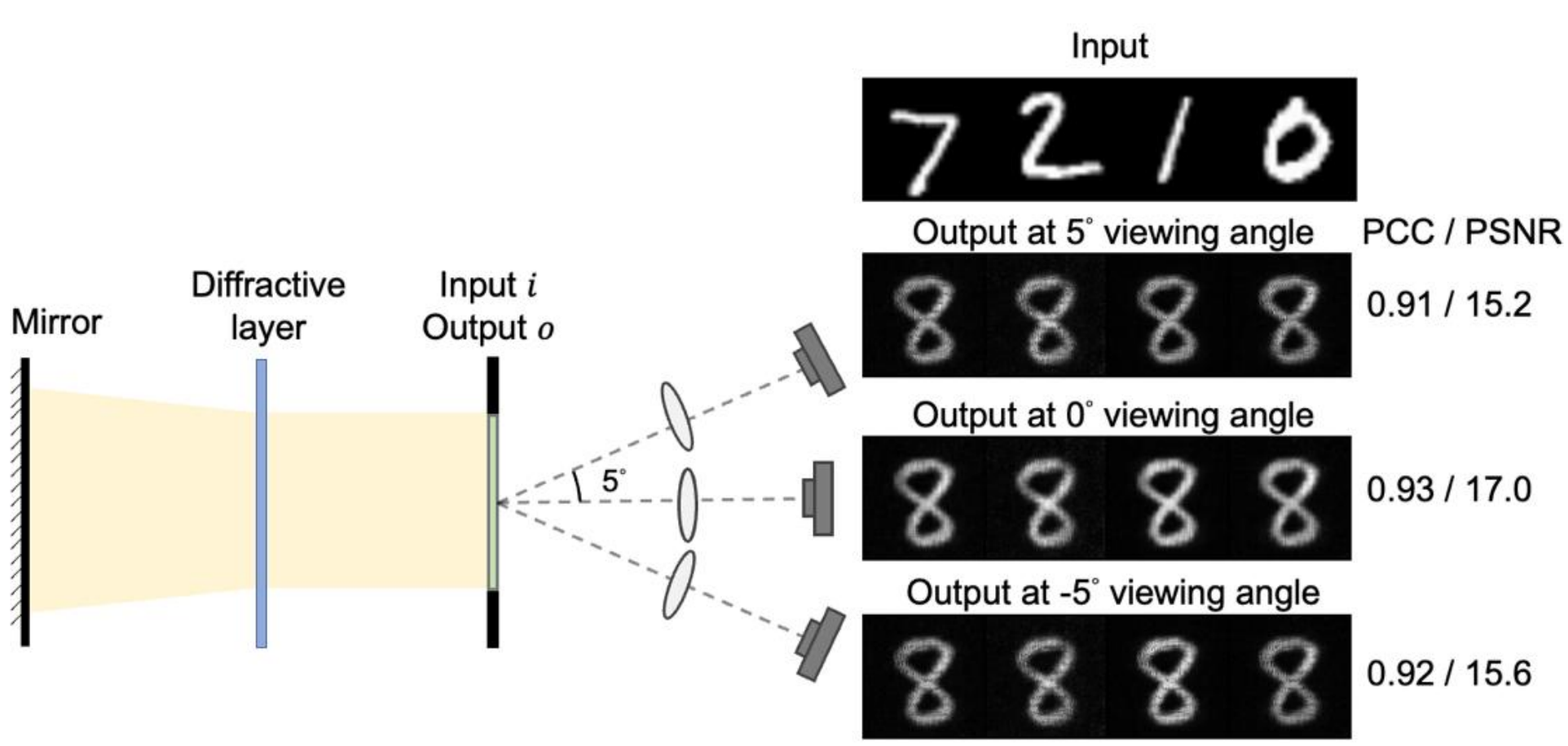

Accordingly, we have revised the **Discussion section** of the manuscript to discuss these analyses and the newly added ***Supplementary Fig. S11***:

> *"…Additionally, we demonstrate that the lying mirror maintains its functionality across multiple viewing angles. To showcase this, we trained a lying mirror using only 0° viewing angle, as we did in earlier designs, and blindly tested its generalization at ±5° viewing angles. As shown in **Supplementary Fig. S11**, the same deceptive output image was observed across a range of viewing angles (±5°), even when the input images varied. Output images at ±5° viewing angles revealed a similar performance as the 0° viewing angle, quantified by the PSNR and PCC values in each viewing angle; **Supplementary Fig. S11** showed that the mean PSNR and PCC values at ±5° viewing angles only decreased by ~8.2% and ~1.6%, respectively, compared to the 0° viewing angle. While these results confirm that the lying mirror preserves its deceptive image transformation capability under different viewing angles, the range of viewing angles can be further enhanced by taking into account the desired coverage of angles in the training phase of the lying mirror, similar to our multi-wavelength lying mirror designs outlined earlier."*

Furthermore, as detailed in our manuscript, the lying mirror system exhibits strong resilience to various perturbations and demonstrates effective wavelength multiplexing as well as reliable performance under partially coherent illumination. These capabilities are validated and presented in <u>Figs. 4, 5, 8, and 9</u>, as well as <u>Supplementary Figs. S7 and S10</u>, highlighting the practical relevance, robustness, and versatility of our approach.

We trust these clarifications effectively address the concerns and reaffirm the originality and significance of our contributions.

In addition to these revisions outlined above, we also attached at the end of this document our former Response Letter, for completeness, summarizing our responses to the 5 technical questions that Reviewer 3 raised in the earlier round of the reviews.

We sincerely appreciate the opportunity to further clarify the remaining points of concern and thank the reviewers for helping us improve our manuscript.

Reviewer #4:

I co-reviewed this manuscript with one of the reviewers who provided the listed reports. This is part of the Nature Communications initiative to facilitate training in peer review and to provide appropriate recognition for Early Career Researchers who co-review manuscripts.
-- We sincerely thank the reviewer for co-reviewing this manuscript. We greatly value the thoughtful suggestions provided, which have been instrumental in improving the quality of our work.

We sincerely thank the referees for their comments and for the constructive feedback that we have received on our manuscript "***Lying mirror using structured surfaces***" submitted to *Nature Communications*.

Reviewer #5:

I recommend acceptance of the re-revised manuscript.

We sincerely thank the reviewer for recommending acceptance of our re-revised manuscript. We are grateful for the reviewers' constructive feedback and valuable time throughout the review process.
